# ^19^F-NMR Diastereotopic Signals in Two *N*-CHF_2_ Derivatives of (4*S*,7*R*)-7,8,8-Trimethyl-4,5,6,7-tetrahydro-4,7-methano-2*H*-indazole †

**DOI:** 10.3390/molecules22112003

**Published:** 2017-11-17

**Authors:** Diana García-Pérez, Concepción López, Rosa M. Claramunt, Ibon Alkorta, José Elguero

**Affiliations:** 1 Departamento de Química Orgánica y Bio-Orgánica, Facultad de Ciencias, UNED, Senda del Rey 9, E-28040 Madrid, Spain; diana2488@hotmail.com (D.G.-P.); rclaramunt@ccia.uned.es (R.M.C.); 2 Instituto de Química Médica, CSIC, Juan de la Cierva 3, E-28006 Madrid, Spain; iqmbe17@iqm.csic.es

**Keywords:** ^19^F-NMR, diastereotopic, anisochrony, pyrazoles, indazoles, GIAO, B3LYP, PCM

## Abstract

In this paper, we report the anisochrony of the fluorine atoms of a CHF_2_ group when linked to a pyrazole ring. The pyrazole is part of (4*S*,7*R*)-7,8,8-trimethyl-4,5,6,7-tetrahydro-4,7-methano-2*H*-indazole also known as (4*S*,7*R*)-campho[2,3-*c*]pyrazole, which has two stereogenic centers. Gauge-Independent Atomic Orbital (GIAO)/Becke, 3-parameter, Lee-Yang-Parr (B3LYP)/6-311++G(d,f) calculated ^19^F chemical shifts of the minimum energy conformations satisfactorily agree with the experimental data. The energy differences between minima need to consider solvent effects (continuum model) to be satisfactorily reproduced.

## 1. Introduction

Anisochrony in NMR is observed when a prochiral group is linked to a molecule possessing a stereogenic center. In these conditions, the studied nuclei became diastereotopic [1,2,3,4]. In the majority of cases, the literature reports concern ^1^H-NMR and often the protons of CH_2_X groups (e.g., benzyl groups) [5,6]. The phenomenon can be observed on the methyl groups of Me_2_X substituents (e.g., isopropyl groups), with both ^1^H- and ^13^C-NMR [7]. Much less common is the observation of the anisochrony of phenyl substituents in CPh_2_X groups, also with ^1^H- and ^13^C-NMR [8,9].

The observation of diastereotopic signals for other nuclei have been reported less often, but, for instance ^31^P [10,11,12,13,14,15,16,17,18] is much more common than for ^15^N, where only one example has been described [19]. Other seldom-explored nuclei are ^2^H [20], ^3^H [21], ^7^Li [22], and ^17^O [23].

In the present paper, we present our results concerning the observation of ^19^F diastereotopic signals. In 1957, anisochronous signals were already observed for F_2_BrC–C*HBrPh, before the phenomenon was clearly understood [24]. Since then, the phenomenon has been repeatedly described, mainly for CHF_2_ groups [25,26,27], but also for CRF_2_ groups [28,29] as well as CRAr_2_ (Ar = *meta* and *para* substituted with F atoms) and CR(CH_2_F)_2_ [30].

**Figure 1:**
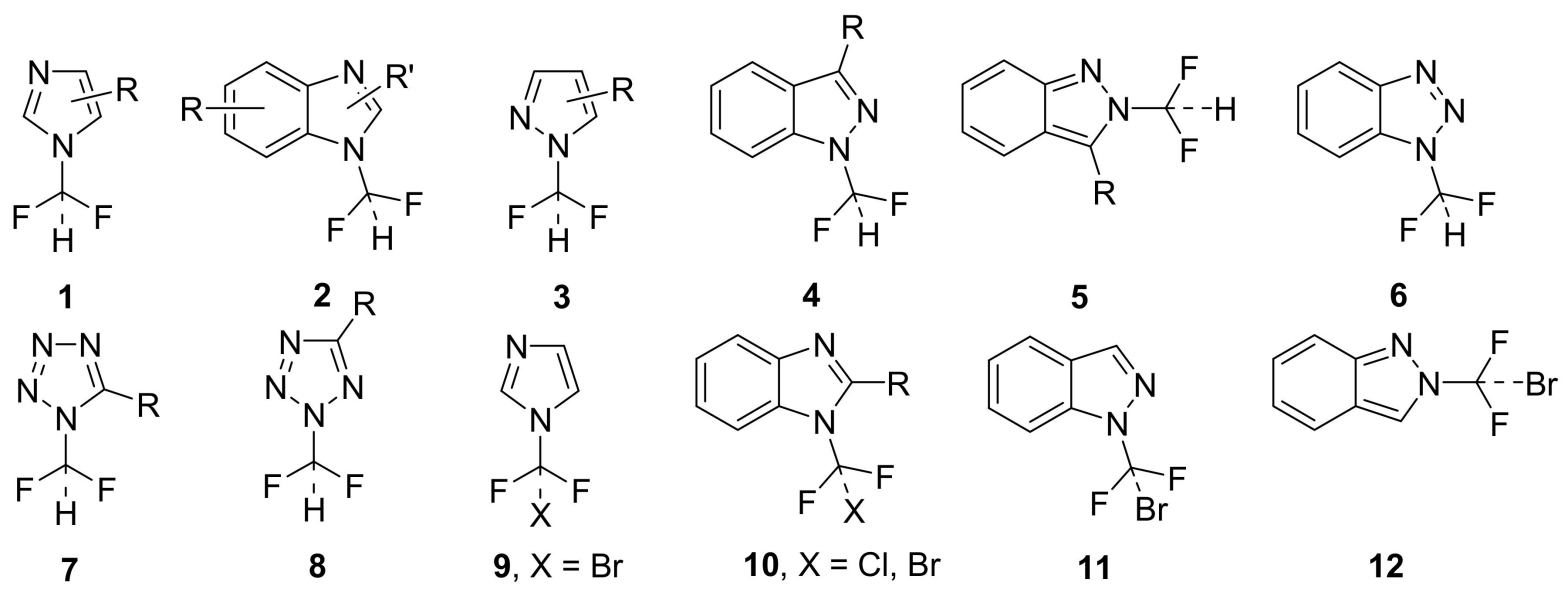
*N*-CHF_2_, *N*-CClF_2_, and *N*-CBrF_2_ azoles and benzazoles.

None of the examples reported in the preceding paragraph concern a chiral molecule containing an *N*-CHF_2_ substituent. There are many examples of azoles bearing a *C*-CHF_2_ substituent, mainly in agrochemistry [31,32,33], the field of *N*-CHF_2_ and *N*-CRF_2_ azoles is less studied although there are several articles dealing with the structures presented in Figure 1.

Imidazoles **1** and benzimidazoles **2** [34,35,36], pyrazoles **3** [37,38], indazoles **4** and **5** [35,39], benzotriazole **6** [34,35,36] were reported. Related compounds **9**–**12** with CXF_2_ substituents are described in reference [40].

**Scheme 1:**
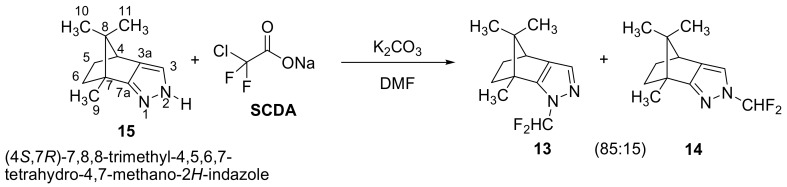
Synthesis of the *N*-difluoromethyl derivatives **13** and **14** of (4*S*,7*R*)-campho[2,3-*c*]pyrazole. SCDA: sodium chlorodifluoroacetate.

The compounds we have prepared (Scheme 1) and studied, **13** and **14**, are derivatives of (4*S*,7*R*)-7,8,8-trimethyl-4,5,6,7-tetrahydro-4,7-methano-2*H*-indazole also known as (4*S*,7*R*)-campho[2,3-*c*]pyrazole, a compound we have previously investigated [41,42,43,44].

## 2. Results and Discussion

### 2.1. Chemistry

As indicated in Scheme 1, compounds **13** and **14** were prepared for the first time by direct difluoromethylation of camphopyrazole **15** with sodium chlorodifluoroacetate (SCDA) [45], according to the Mehta and Greaney conditions [46] or by adding a phase transfer catalyst [47], in both cases using *N*,*N*′-dimethylformamide as solvent and K_2_CO_3_ as base. Both isomers were obtained in an 85:15 ratio (see Experimental Section). The only other paper where the *N*-substitution of **15** was reported (with 1,2-dichloroethane) yielded a 50:50 mixture of both isomers [48]. The structure elucidation of compounds **13** and **14** was based on the close correlation of the ^13^C chemical shifts of the pyrazole ring with those of a reference compound [48].

### 2.2. NMR Spectroscopy

**Figure 2:**
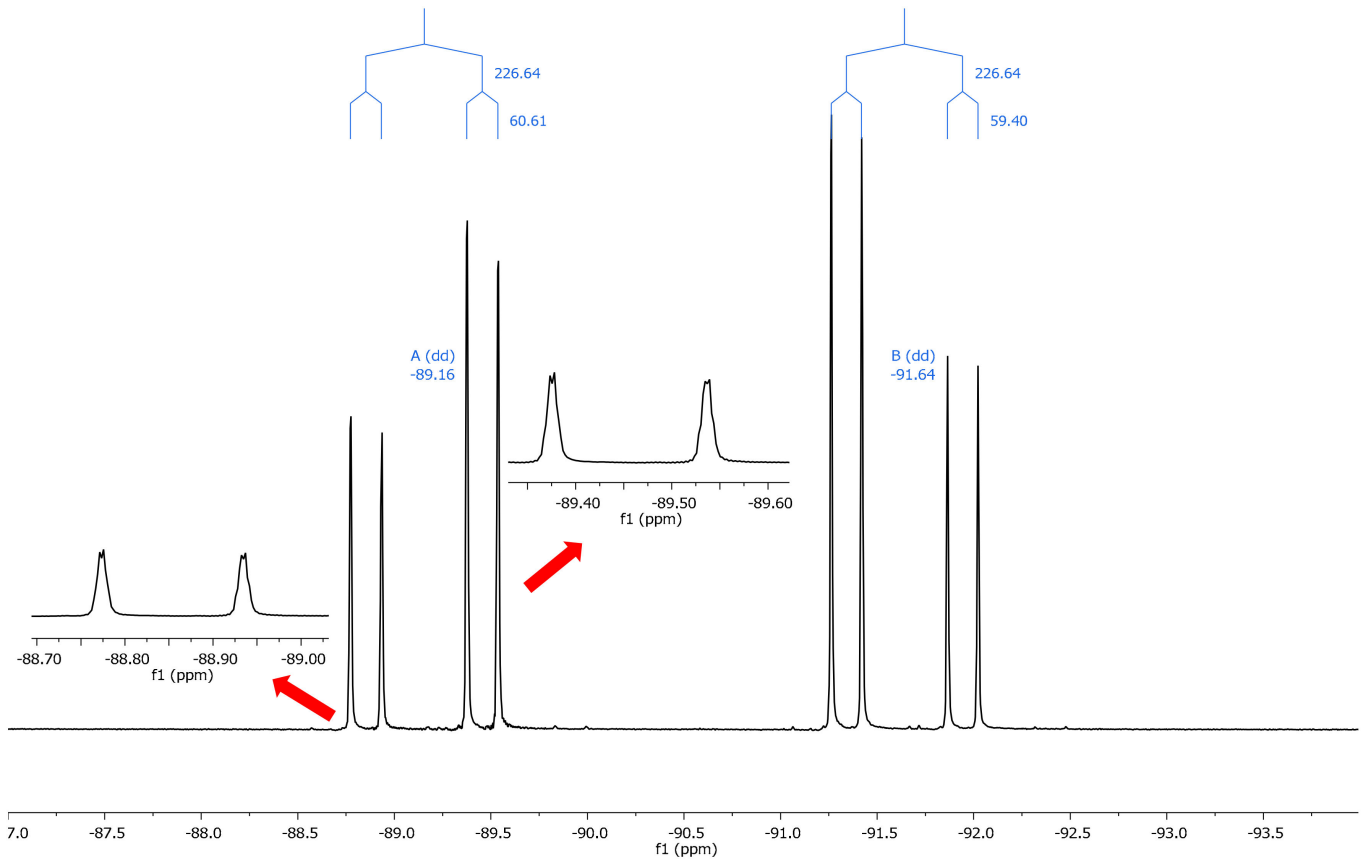
^19^F-NMR spectrum of **13** in CDCl_3_ at 300 K with signals at −89.16 ppm (ddd, ^2^*J*_F_ = 226.6, ^2^*J*_H_ = 60.6, ^6^*J*_H_ = 1.4), and −91.64 (dd, ^2^*J*_F_ = 226.6, ^2^*J*_H_ = 59.4); the red arrows correspond to the amplification of the left and right side of the signal at −89.16 ppm.

**Figure 3:**
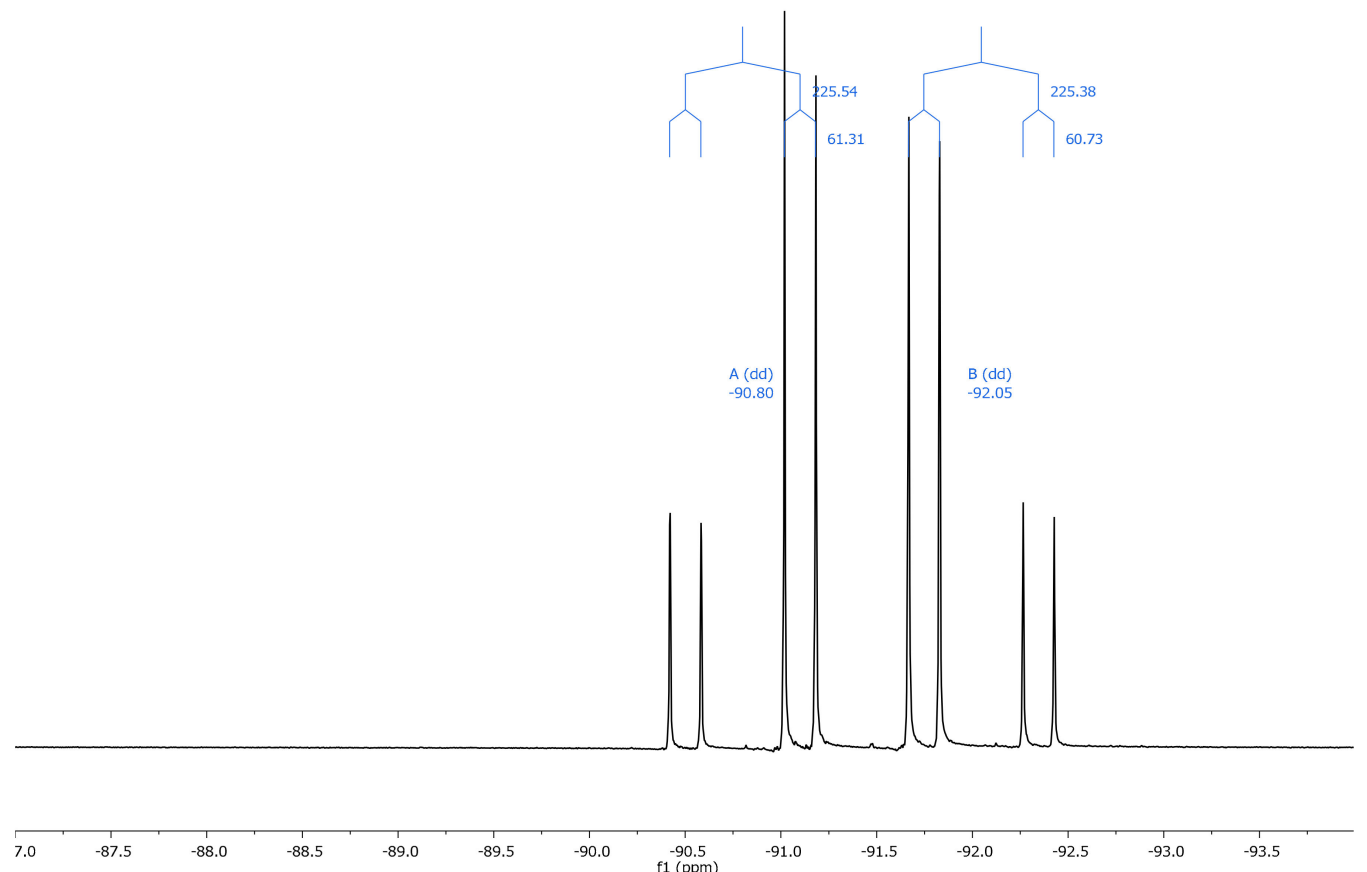
^19^F-NMR spectrum of **14** in CDCl_3_ at 300 K with signals at −90.80 ppm (dd, ^2^*J*_F_ = 225.5, ^2^*J*_H_ = 61.3), and −92.05 (dd, ^2^*J*_F_ = 225.4, ^2^*J*_H_ = 60.7).

In both configurational isomers, the fluorine atoms are diastereotopic, and two distinct signals were observed for each one. From the spectra (Figure 2 and Figure 3 and data given in Supplementary Materials), ^2^*J*(^1^H-^19^F) and ^2^*J*(^19^F-^19^F) coupling constants can be measured.

The ^2^*J*_FF_ SSCC (spin-spin coupling constant) in F-C-F compounds is very sensitive to structural aspects, especially the C atom hybridization; for sp^3^ carbons range between 3.5 and 340 Hz [49]. There are no ^2^*J*_FF_ values published for *N*-azolyl derivatives, and thus the values we have measured (about 225 Hz) are the only representatives of this kind of compound.

In ^1^H-NMR (see experimental part and Supplementary Material), the most interesting information concerning the CHF_2_ group where when the anisochrony is larger (compound **13**) the two ^2^*J*_HF_ couplings are different and when the anisochrony is smaller (compound **14**) they are identical. Moreover, the signal of the 9-CH_3_ group in compound **13** shows a long-distance ^6^*J*_HF_ coupling of 1.4 Hz (also measured in the ^19^F-NMR spectrum, see Figure 2); in compound **14**, this coupling is not observed due to the additional bond (it would be a ^7^*J*_HF_).

### 2.3. Computational Results

**Figure 4:**
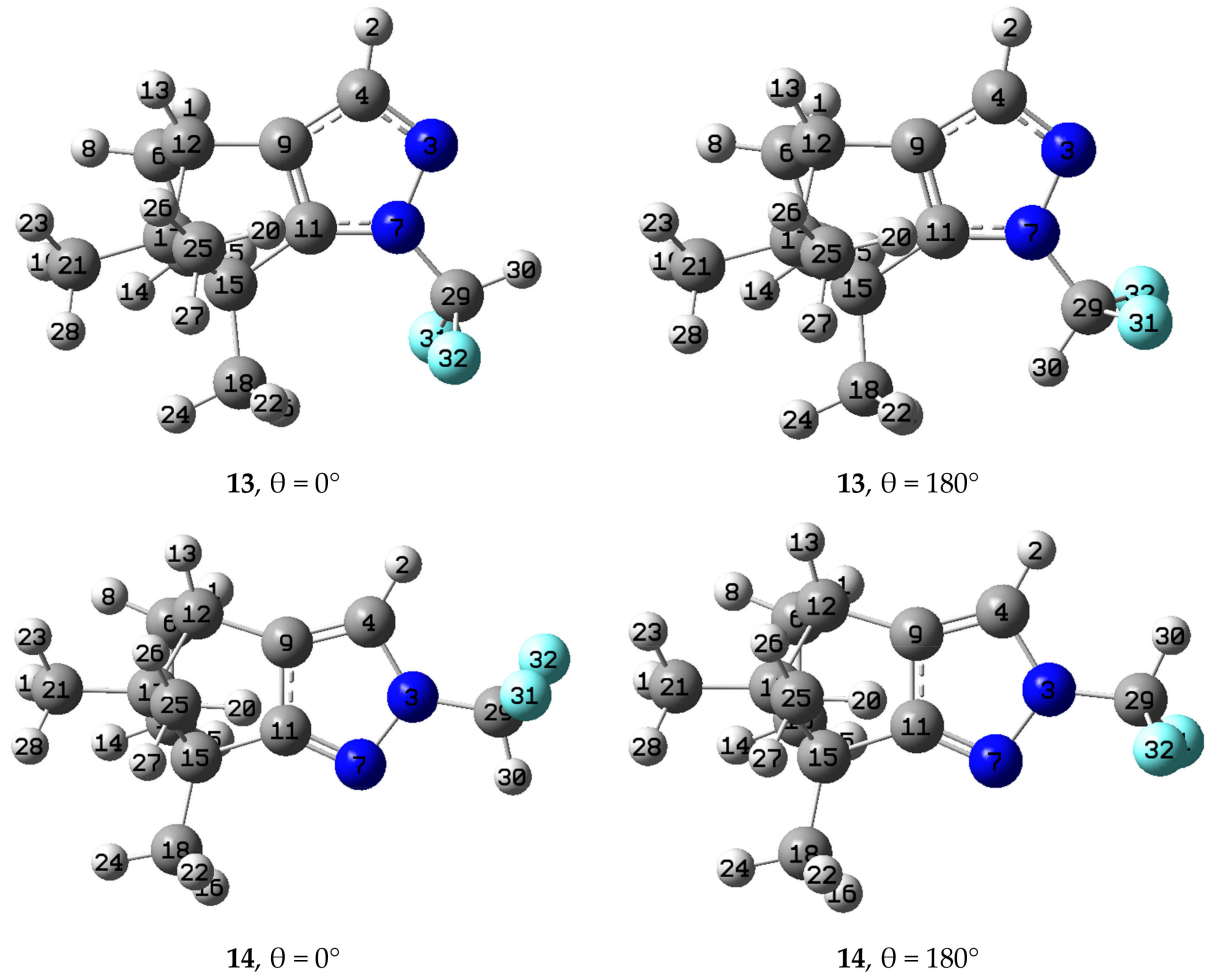
The four minima.

We have calculated the energy of compounds **13** and **14** as a function of the torsion angle θ about the *N*-(CHF_2_) bond (defined as H-C-N1-N2, 30-29-7-3 or 30-29-3-7). There are two minima (0 imaginary frequencies)—one near 0° and the other near 180° (Figure 4).

According to the calculations, the 2-substituted isomer **14** is more stable than the 1-substituted isomer **13** by 10.8 kJ·mol^–1^ (both in their minima; i.e., having 0 imaginary frequencies). Note that in camphopyrazole, tautomer 2*H* is more stable than tautomer 1*H* [41,43,44] due to the Mills–Nixon effect [50,51]; once again, tautomerism and isomerism behave similarly.

**Figure 5:**
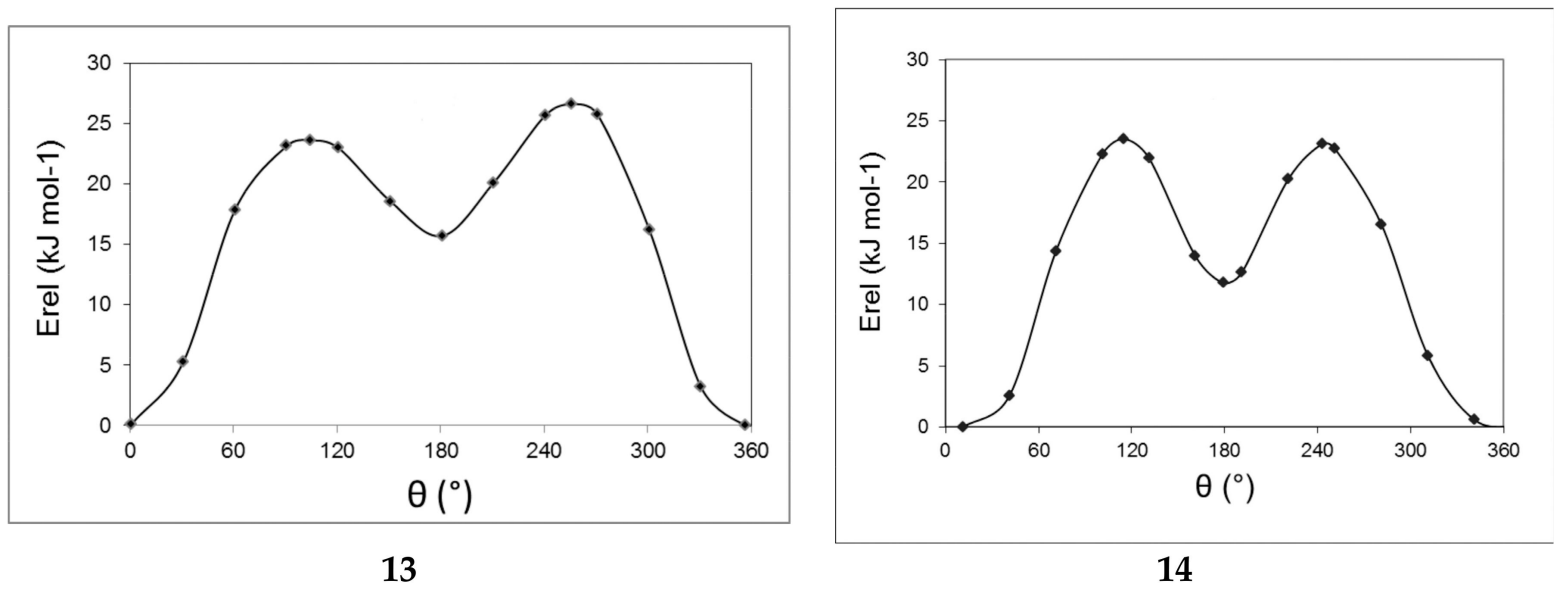
Energy profiles in kJ·mol^–1^ vs. the dihedral angle θ.

When the energy was calculated as a function of the torsion angle θ about the *N*-(CHF_2_) bond, in both cases, the minimum energy conformation corresponds to θ = 0°; i.e., the H atom of the CHF_2_ group eclipsing the “pyridine-like” N atom of pyrazole, the so-called *syn*-periplanar conformation (Figure 5). The difference between the 0° and the 180° minima are for **13** 15.7 kJ·mol^–1^ and for **14** 11.8 kJ·mol^–1^, and the transition states are for **13** 23.6 (θ = 104.4°) and 26.6 kJ·mol^–1^ (θ = 255.8°) and for **14** 23.5 (θ = 114.9°) and 23.1 kJ·mol^–1^ (θ = 242.9°).

This conformational preference can most probably be explained by the dominance of vicinal hyperconjugation, with electron donation from the electron-rich sigma N-N bonding orbital into both of the very electron deficient vicinal C-F anti-bonding orbitals [52,53,54,55].

A natural bond orbital (NBO) analysis shows that the energetic difference between the conformations minima at 0° and 180° can be explained based on the stabilization due to the sum of the charge transfer between the lone pair of the pyridine-like nitrogen and the σ* C-H bond and between the σ N-N and the σ* C-F bonds. This stabilization amount is 6.6 kJ·mol^–1^ in the minima at 0° of **13** and **14**, while in the minima at 180° it is between 1.1 and 1.0 kJ·mol^–1^, respectively.

Gauge-Independent Atomic Orbital (GIAO) calculated parameters (absolute shieldings) accounted for the experimental results obtained by multinuclear NMR (^1^H, ^13^C, ^15^N and ^19^F) (see Supplementary Materials). We will focus on the ^19^F chemical shifts (Table 1).

The four experimental values (−91.6, −89.2, −92.0, −90.8, ppm) are close to the calculated ones for **13** (0°) (−93.2, −89.7 ppm) and for **14** (0°) (−92.8, −88.7 ppm) than for the 180° assignment (−98.5, −90.1, −97.4, −85.7 ppm). Assuming the simplification that only the two minima contribute to the experimental values, a simple interpolation of the type Exp = a × (Calc. abs minima) + (1–a) × (Calc. second minima) lead to **13** = 91.8% of conformer θ ≈ 0° and 8.2% of conformer θ ≈ 180°, and **14** = 81.6% of conformer θ ≈ 0° and 18.4% of conformer θ ≈ 180°. This corresponds at 298.15 K to −6.0 and −3.7 kJ·mol^−1^, respectively—lower than the calculated differences between both rotamers, but of the same sign. To see if the inclusion of solvent effects improves the agreement, we calculated the differences of energy between minima in CHCl_3_ (Polarizable continuum model, PCM) obtaining for **13** and **14**, −7.6 and −5.1 kJ·mol^−1^, respectively—much closer to the experimental results (the TS have very close values: 19.8 and 19.2 kJ·mol^−1^); the solvent slightly modifies the geometries, see θ values in Table 2.

We have calculated the chemical shifts in CHCl_3_, obtaining the values reported in Table 2. With these values, we have calculated that the difference of energies for **13** and **14** are −4.9 and −4.3 kJ·mol^−1^, respectively, comparable to those obtained for the gas phase (−6.0 and −3.7 kJ·mol^−1^) to be compared with −7.6 and −5.1 kJ·mol^−1^.

We have also calculated the ^13^C chemical shifts of the three carbon atoms of the pyrazole ring (C3, C3a, C7a named C4, C9, and C11 in Figure 3). The results are reported in Table 3 and correlates well with the experimental carbon signal shifts, and aided the assignment of the pyrazole ring carbons.

## 3. Experimental Section

### 3.1. Chemistry

General

All chemicals cited in the synthetic procedure are commercial compounds. Melting points were determined by differential scanning calorimetry (DSC) with a SEIKO DSC 220 C connected to a model SSC5200H disk station. Thermograms (sample size 0.003–0.005 g) were recorded with a scan rate of 5.0 °C. Column chromatography was performed on silica gel 60 (Merck KGaA, Darmstadt, Germany), 70–230 mesh), and elemental analyses using a Perkin-Elmer 240 apparatus (Madrid, Spain).

Preparation of (4*S*,7*R*)-1-(Difluoromethyl)-7,8,8-trimethyl-4,5,6,7-tetrahydro-4,7-methano-1*H*-indazole (**13**) and (4*S*,7*R*)-2-(Difluoromethyl)-7,8,8-trimethyl-4,5,6,7-tetrahydro-4,7-methano-1*H* -indazole (**14**).

Procedure A from Ref. [46]. Into a 100-mL round-bottom three-necked flask equipped with reflux condenser and magnetic stirring, 2 equivalents of sodium chlorodifluoroacetate (SCDA) and 1.5 equivalents of the base (K_2_CO_3_) were introduced. The vacuum was established for 15 min and then purged with argon for another 15 min (this process was repeated three times). Six milliliters of *N*,*N*-dimethylformamide (DMF) was added slowly with stirring and under an argon stream, and then 1 equivalent of (4*S*,7*R*)-7,8,8-trimethyl-4,5,6,7-tetrahydro-4,7-methano-2*H*-indazole (**15**) dissolved in 2 mL of DMF was added from an addition funnel over 15 min. The flask was immersed in a silicone bath previously heated to 100 °C and left stirring for 8 h. To control the temperature, a thermometer was used which was connected to the heating plate and immersed in the silicone oil bath. After the reaction time was completed, it was cooled to room temperature and EtOAc (15 mL) and water (15 mL) were added to the mixture. The organic fraction was washed with brine, and the aqueous fraction was extracted with EtOAc. The organic fractions were combined, dried over anhydrous MgSO_4_, and the solvent evaporated off. The yield of the reaction crude—in which both isomers are present in a ratio (85% of **13**: 15% of **14**)—is quantitative. The purification was carried out by column chromatography using dichloromethane/hexane (1:1) as eluent. Compound **14** was eluted first.

Procedure B from Ref [47]. Into a 100-mL round-bottom flask equipped with reflux condenser and magnetic stirring, 2 equivalents of SCDA, 3 equivalents of the base (K_2_CO_3_), 1 equivalent of (4*S*,7*R*)-7,8,8-trimethyl-4,5,6,7-tetrahydro-4,7-methano-2*H*-indazole (**15**), and 0.3 equivalents of tetraethylammonium bromide (TEAB) were dissolved in 10 mL of DMF and the mixture was stirred at 100 °C for 3 h. The resulting mixture was poured into water and extracted with EtOAc, the organic extract containing again an 85:15 mixture of both isomers (overall yield 90%) was treated as previously described in procedure A.

*(4S,7R)-1-(Difluoromethyl)-7,8,8-trimethyl-4,5,6,7-tetrahydro-4,7-methano-1H-indazole* (**13**). m.p.: 45.4 °C; ^1^H-NMR: (400.13 MHz, CDCl_3_) δ = 7.27 (s, H_3_), 7.14 (dd, ^2^*J*_F_ = 59.5, ^2^*J*_F_ = 60.5, CHF_2_), 2.81 (d, ^3^*J* = 3.8), 2.05 (cm, H_5ec_), 1.03 (cm, H_5ax_), 1.81(cm, H_6ec_), 1.18 (cm, H_6ax_), 1.37 (dd, ^6^*J*_F_ = 1.4, CH_3_-9), 0.92 (s, CH_3_-10), 0.77 (s, CH_3_-11); ^13^C-NMR: (100.61 MHz, CDCl_3_) δ = 153.6 (dd, ^3^*J*_F_ = 1.6, C7a), 134.3 (dd, ^4^*J*_F_ = 2.3, C3), 132.1 (C3a), 111.6 (dd, ^1^*J*_F_ = 246.0, ^1^*J*_F_ = 248.7, CHF_2_), 63.2 (C8), 53.7 (C7), 47,6 (C4), 33.0 (C6), 27.4 (C5), 20.1 (CH_3_-11), 19.5 (CH_3_-10), 11.6 (dd, ^5^*J*_F_ = ^5^*J*_F_ = 1.4, CH_3_-9); ^19^F NMR: (376.50 MHz, CDCl_3_) δ = −89.16 (ddd, ^2^*J*_F_ = 226.6, ^2^*J*_H_ = 60.6, ^6^*J*_H_ = 1.4), −91.64 (dd, ^2^*J*_F_ = 226.6, ^2^*J*_H_ = 59.4); ^15^N-NMR: (40.54 MHz, CDCl_3_) δ = −177.4 (dd, ^2^*J*_F_ = ^2^*J*_F_ = 27.9, N1), −79.9 (N2). Anal. calcd. for C_12_H_16_F_2_N_2_: C 63.70, H 7.13, N 12.38. Found: C 63.45, H 7.45, N 12.13.

*(4S,7R)-2-(Difluoromethyl)-7,8,8-trimethyl-4,5,6,7-tetrahydro-4,7-methano-1H-indazole* (**14**). m.p.: 40.7 °C; ^1^H-NMR: (400.13 MHz, CDCl_3_) δ = 7.28 (s, H_3_), 7.11 (dd, ^2^*J*_F_ = ^2^*J*_F_ = 60.9, CHF_2_), 2.79 (d, ^3^*J* = 4.1), 2.10 (cm, H_5ec_), 1.22 (cm, H_5ax_), 1.88 (cm, H_6ec_), 1.35 (cm, H_6ax_), 1.29 (s, CH_3_-9), 0.97 (s, CH_3_-10)), 0.65 (s, CH_3_-11); ^13^C-NMR: (100.61 MHz, CDCl_3_) δ = 169.1 (dd, ^4^*J*_F_ = ^4^*J*_F_ = 2.2, C7a), 130.2 (C3a), 117.9 (C3), 111.2 (dd, ^1^*J*_F_ = 246.4, ^1^*J*_F_ = 246.5, CHF_2_), 60.4 (C8), 50.1 (C7), 46.9 (C4), 33.3 (C6), 27.2 (C5), 20.4 (CH_3_-11), 18.9 (CH_3_-10), 10.4 (CH_3_-9); ^19^F-NMR: (376.50 MHz, CDCl_3_) δ = −90.80 (dd, ^2^*J*_F_ = 225.5, ^2^*J*_H_ = 61.3), −92.05 (dd, ^2^*J*_F_ = 225.4, ^2^*J*_H_ = 60.7); ^15^N-NMR: (40.54 MHz, CDCl_3_) δ = −177.2 (dd, ^2^*J*_F_ = ^2^*J*_F_ = 24.9, N2), N1 not detected. Anal. calcd. for C_12_H_16_F_2_N_2_: C 63.70, H 7.13, N 12.38. Found: C 63.37, H 7.48, N 11.98.

### 3.2. NMR

NMR spectra were recorded on a Bruker (Bruker Biospin GmbH, Rheinstetten, Germany) DRX 400 (9.4 Tesla, 400.13 MHz for ^1^H, 100.61 MHz for ^13^C and 40.54 MHz for ^15^N using a 5-mm inverse-detection H-X probe equipped with a z-gradient coil, at 300 K. Chemical shifts (δ in ppm) are given from internal solvent, CDCl_3_ 7.26 for ^1^H and 77.0 for ^13^C and for ^15^N, nitromethane (0.00) was used as external reference. Signals were characterized as s (singlet), d (doublet), and cm (complex multiplet) and the *J* coupling constants are given in Hz.

Typical parameters for ^1^H-NMR spectra were spectral width 4800 Hz and pulse width 9.5 μs at an attenuation level of 0 dB. Typical parameters for ^13^C-NMR spectra were spectral width 21 kHz, pulse width 12.5 μs, at an attenuation level of −6 dB and relaxation delay 2 s, WALTZ-16 was used for broadband proton decoupling; the Free Induction Decays (FIDs) were multiplied by an exponential weighting (lb = 1 Hz) before Fourier transformation.

Inverse proton detected heteronuclear shift correlation spectra, (^1^H-^13^C) gs-HMQC, and (^1^H-^13^C) gs-HMBC were acquired and processed using standard Bruker NMR software and in non-phase-sensitive mode. Gradient selection was achieved through a 5% sine truncated shaped pulse gradient of 1 ms.

Selected parameters for (^1^H-^13^C) gs-HMQC and (^1^H-^13^C) gs-HMBC spectra were spectral width 4800 Hz for ^1^H and 20.5 kHz for ^13^C, 1024 × 256 data set, number of scans two (gs-HMQC) or four (gs-HMBC) and relaxation delay 1 s. The FIDs were processed using zero filling in the *F_1_* domain and a sine-bell window function in both dimensions was applied prior to Fourier transformation. In the gs-HMQC experiments, Globally Optimized Alternating Phase Rectangular Pulse (GARP) modulation of ^13^C was used for decoupling. Selected parameters for (^1^H-^15^N) gs-HMQC, and (^1^H-^15^N) gs-HMBC spectra were spectral width 3500 Hz for ^1^H and 12.5 kHz for ^15^N, 1024 × 256 data set, number of scans four, relaxation delay 1 s, 37–60 ms delay for evolution of the ^15^N-^1^H long-range coupling. The FIDs were processed using zero filling in the *F*_1_ domain and a sine-bell window function in both dimensions was applied prior to Fourier transformation.

^19^F-NMR spectra were recorded on the same spectrometer (376.50 for ^19^F) using a 5 mm Quattro Nucleus Probe (QNP) direct-detection probehead equipped with a z-gradient coil, at 300 K. Chemical shifts (δ in ppm) are given from CFCl_3_ as external reference (one drop of CFCl_3_ in CDCl_3_ (0.00)). Typical parameters for ^19^F NMR spectra were spectral width of 55 kHz, pulse width of 13.75 µs at attenuation level of −6 dB and relaxation delay of 1 s. WALTZ-16 was used for broadband proton decoupling ^19^F{^1^H}, the FIDS were multiplied by an exponential weighting (lb = 1 Hz) before Fourier transformation.

### 3.3. Computational Details

Calculations were carried out at the B3LYP/6-311++G(d,p) level [56,57]. Subsequent frequency calculations verify that the structures obtained correspond to energetic minima (imaginary frequencies = 0) or to transition states (imaginary frequencies = 1). In the optimization process, the 0° and 180° angles get slightly modified (Table 1 and Table 2). These resulting geometries have been used for the calculation of the absolute chemical shieldings with the GIAO method [58,59]. Solvent effects were calculated within the PCM approximation (continuum model) [60,61,62]. All the calculations have been performed with the Gaussian-09 package [63].

Equations (1)–(4) [64,65,66] have been used to transform absolute shieldings into chemical shifts:

δ^1^H = 31.0 − 0.97 × σ^1^H, (reference TMS, 0.00 ppm) (1)

δ^13^C = 175.7 − 0.963 × σ^13^C, (reference TMS, 0.00 ppm) (2)

δ^15^N = −152.0 − 0.946 × σ^15^N, (reference TMS, 0.00 ppm) (3)

δ^19^F = 162.1 − 0.959 × σ^19^F, (reference CFCl_3_, 0.00 ppm) (4)

The natural bond orbital (NBO) method [67] has been used to obtain the stabilizing charge-transfer interactions in complexes using the NBO-6 program [68].

## 4. Conclusions

In summary, we have found a new and original example of diastereotopic fluorine atoms, measured two values of ^2^*J*_FF_ in an original environment and successfully carried out GIAO/B3LYP/6-311++G(d,p) calculations of ^19^F chemical shifts that agree with the calculated energies of the two minima of the potential energy curve when solvent was taken into account.

## Figures and Tables

**Table 1:** Calculated (gas phase) and experimental ^19^F-NMR chemical shifts (CDCl_3_).

Comp.	θ (°)	^19^F (31)	^19^F (32)	Δδ (31–32)	^19^F (a)	^19^F (b)	Δδ (a–b) ^a^
	Calculated Values				Experimental Values		
**13**	–3.6	–93.18	–89.73	−3.45	–91.64	–89.16	−2.48
**13**	–179.3	–90.07	–98.52	+8.45			
**14**	11.1	–92.83	–88.66	–4.17	–92.05	–90.80	–1.25
**14**	179.3	–85.73	–97.40	+11.67			

^a^ The sign is arbitrary because the assignment of **a** and **b** is also arbitrary.

**Table 2:** Calculated (CHCl_3_) and experimental ^19^F-NMR chemical shifts (CDCl_3_).

Comp.	θ (°)	^19^F (31)	^19^F (32)	Δδ (31–32)	^19^F (a)	^19^F (b)	Δδ (a–b) ^a^
	Calculated Values				Experimental Values		
**13**	−4.0	−94.07	−90.23	−3.84	−91.64	−89.16	−2.48
**13**	−179.5	−91.97	−99.34	+7.37			
**14**	11.2	−94.21	−90.68	−3.53	−92.05	−90.80	−1.25
**14**	179.4	−86.35	−97.99	+11.64			

^a^ The sign is arbitrary because the assignment of **a** and **b** is also arbitrary.

**Table 3:** Comparison of experimental and calculated ^13^C chemical shifts.

Comp.	13 exp. CDCl_3_	13 calc. Gas	13 calc. CHCl_3_	14 exp. CDCl_3_	14 calc. Gas	14 calc. CHCl_3_
C3 (C4)	134.3	133.2	134.4	117.9	117.3	118.2
C3a (C9)	132.1	133.0	133.9	130.2	132.3	133.6
C7a (C11)	153.6	153.7	155.2	169.1	167.5	169.3

## Supplementary Materials

Supplementary materials are available online: Tables S1–S3 and Figures S1–S14.
