# Supplementary material for: 19F-NMR Diastereotopic Signals in Two N-CHF2 Derivatives of (4S,7R)-7,8,8-Trimethyl-4,5,6,7-tetrahydro-4,7-methano-2H-indazole
Source: Molecules. 2017 Nov 17;22(11):2003. doi: 10.3390/molecules22112003 (PMC6150283; doi:10.3390/molecules22112003)
Supplement: Supplementary file 1 [file molecules-22-02003-s001.pdf]

SUPPLEMENTARY MATERIALS

^{19}F NMR Diastereotopic Signals in Two *N*-CHF_2 derivatives of (4*S*,7*R*)- 7,8,8-trimethyl-4,5,6,7-tetrahydro-4,7-methano-2*H*-indazole

Diana García-Pérez[1], Concepción López[1,*], Rosa M. Claramunt[1], Ibon Alkorta[2,*] and José Elguero

1 Dpto. Química Orgánica y Bio-Orgánica, Facultad de Ciencias, UNED, Senda del Rey 9, E-28040 Madrid, Spain
2 Instituto de Química Médica, CSIC, Juan de la Cierva 3, E-28006 Madrid, Spain
* Correspondence: clopez@ccia.uned.es (C.L.); ibon@iqm.csic.es (I.A.)

CONTENTS

NMR

- **Table S1:** ^{1}H chemical shifts and spin-spin coupling constants (*J*, Hz) in $CDCl_3$ of compounds **13-14** at 300 K.
- **Table S2:** ^{13}C chemical shifts and spin-spin coupling constants (*J*, Hz) with fluorine in $CDCl_3$ of compounds **13-14** at 300 K.
- **Table S3:** ^{15}N and ^{19}F chemical shifts and spin-spin coupling constants (*J*, Hz) in $CDCl_3$ of compounds **13-14** at 300 K.
- **Figures S1-S7:** ^{1}H, ^{13}C, ^{1}H-^{13}C gs-HMQC, ^{1}H-^{13}C gs-HMBC, ^{1}H-^{15}N gs-HMBC, ^{19}F and ^{19}F{^{1}H} NMR spectra of compound **13** in $CDCl_3$ at 300 K
- **Figures S8-S14**: H, ^{13}C, ^{1}H-^{13}C gs-HMQC, ^{1}H-^{13}C gs-HMBC, ^{1}H-^{15}N gs-HMBC, ^{19}F and ^{19}F{^{1}H} NMR spectra of compound **14** in $CDCl_3$ at 300 K .

COMPUTATIONAL RESULTS

- B3LYP/6-311++G(d,p) computational level results in gas phase of the stationary points (minima and TSs).
- Optimized geometry and Electronic energy
- Chemical shielding and shift (ppm)

NMR parameters

NMR spectra were recorded on a Bruker DRX 400 (9.4 Tesla, 400.13 MHz for ^{1}H, 100.61 MHz for ^{13}C and 40.54 MHz for ^{15}N using a 5-mm inverse-detection H-X probe equipped with a *z*-gradient coil, at 300 K. Chemical shifts (δ in ppm) are given from internal solvent, $CDCl_3$ 7.26 for ^{1}H and 77.0 for ^{13}C and for ^{15}N, nitromethane (0.00) was used as external reference. Signals were characterized as s (singlet), d (doublet), and cm (complex multiplet).

Typical parameters for ^{1}H NMR spectra were spectral width 4800 Hz and pulse width 9.5 μs at an attenuation level of 0 dB. Typical parameters for ^{13}C NMR spectra were spectral width 21 kHz, pulse width 12.5 μs at an attenuation level of -6 dB and relaxation delay 2s, WALTZ-16 was used for broadband proton decoupling; the FIDS were multiplied by an exponential weighting (lb=1 Hz) before Fourier transformation.

Inverse proton detected heteronuclear shift correlation spectra, (^{1}H-^{13}C) gs-HMQC and (^{1}H-^{13}C) gs-HMBC, were acquired and processed using standard Bruker NMR software and in nonphase-sensitive mode. Gradient selection was achieved through a 5% sine truncated shaped pulse gradient of 1 ms.

Selected parameters for (^{1}H-^{13}C) gs-HMQC and (^{1}H-^{13}C) gs-HMBC spectra were spectral width 4800 Hz for ^{1}H and 20.5 kHz for ^{13}C, 1024 x 256 data set, number of scans 2 (gs-HMQC) or 4 (gs-HMBC) and relaxation delay 1s. The FIDs were processed using zero filling in the F_1 domain and a sine-bell window function in both dimensions was applied prior to Fourier transformation. In the gs-HMQC experiments, GARP modulation of ^{13}C was used for decoupling. Selected parameters for (^{1}H-^{15}N) gs-HMQC and (^{1}H-^{15}N) gs-HMBC spectra were spectral width 3500 Hz for ^{1}H and 12.5 kHz for ^{15}N, 1024 x 256 data set, number of scans 4, relaxation delay 1s, 37-60 ms delay for evolution of the ^{15}N-^{1}H long-range coupling. The FIDs were processed using zero filling in the F_1 domain and a sine-bell window function in both dimensions was applied prior to Fourier transformation.

^{19}F NMR spectra were recorded on the same spectrometer (376.50 for ^{19}F) using a 5 mm QNP direct-detection probehead equipped with a *z*-gradient coil, at 300 K. Chemical shifts (δ in ppm) are given from $CFCl_3$ as external reference [one drop of $CFCl_3$ in $CDCl_3$ (0.00)]. Typical parameters for ^{19}F NMR spectra were spectral width of 55 kHz, pulse width of 13.75 us at attenuation level of -6 dB and relaxation delay of 1s. WALTZ-16 was used for broadband proton decoupling $^{19}F\{^{1}H\}$, the FIDS were multiplied by an exponential weighting (lb=1 Hz) before Fourier transformation.

Table S1: ^{1}H chemical shifts and spin-spin coupling constants (J, Hz) in $CDCl_3$ of compounds **13-14** at 300 K.

Comp.	CHF_2	H3	H4	H5	H6	Others
**13**	7.14 (dd)<br>$^{2}J_F = 59.5$<br>$^{2}J_F = 60.5$	7.27 (s)	2.81 (d)<br>$^{3}J = 3.8$	1.03^{ax} (cm)<br>2.05^{ec} (cm)	1.18^{ax} (cm)<br>1.81^{ec} (cm)	CH_3–11 = 0.77 (s)<br>CH_3–10 = 0.92 (s)<br>CH_3–9 = 1.37 (dd)<br>$^{6}J_F = {}^{6}J_F$ =1.4
**14**	7.11 (dd)<br>$^{2}J_F = {}^{2}J_F = 60.9$	7.28 (s)	2.79 (d)<br>$^{3}J = 4.1$	1.22^{ax} (cm)<br>2.10^{ec} (cm)	1.35^{ax} (cm)<br>1.88^{ec} (cm)	CH_3–11 = 0.65 (s)<br>CH_3–10 = 0.97 (s)<br>CH_3–9 = 1.29 (s)

Table S2: ^{13}C chemical shifts and spin-spin coupling constants (J, Hz) with fluorine in $CDCl_3$ of compounds **13-14** at 300 K.

Comp.	CHF_2	C3	C3a	C4	C5	C6	C7	C7a	C8
**13**	111.6 (dd)<br>$^{1}J_F = 246.0$<br>$^{1}J_F = 248.7$	134.3 (dd)<br>$^{4}J_F = {}^{4}J_F = 2.3$	132.1	47.6	27.4	33.0	53.7	153.6 (dd)<br>$^{3}J_F = {}^{3}J_F = 1.6$	63.2
	**Others**								
	CH_3 –9 = 11.6 (dd) $^{5}J_F$~$^{5}J_F$ = 1.4; CH_3–10 = 19.5; CH_3–11 = 20.1								
**14**	111.2 (dd)<br>$^{1}J_F = 246.4$<br>$^{1}J_F = 246.5$	117.9	130.2	46.9	27.2	33.3	50.1	169.1 (dd)<br>$^{4}J_F = {}^{4}J_F = 2.2$	60.4
	**Others**								
	CH_3–9 = 10.4; CH_3–10 = 18.9; CH_3–11 = 20.4								

Table S3: ^{15}N and ^{19}F chemical shifts and spin-spin coupling constants (J, Hz) in $CDCl_3$ of compounds **13-14** at 300 K.

Comp.	N1	N2	Fa/Fb	Fa/Fb
**13**	-177.4 (dd)<br>$^{2}J_F = {}^{2}J_F = 27.9$	-79.9	-89.16 (ddd)<br>$^{2}J_F = 226.6$<br>$^{2}J_H = 60.6$<br>$^{6}J_H = 1.4$	-91.64 (dd)<br>$^{2}J_F = 226.6$<br>$^{2}J_H = 59.4$
**14**	n.d.	-177.2(dd)<br>$^{2}J_F = {}^{2}J_F = 24.9$	-90.80 (dd)<br>$^{2}J_F = 225.5$<br>$^{2}J_H = 61.3$	-92.05(dd)<br>$^{2}J_F = 225.4$<br>$^{2}J_H = 60.7$

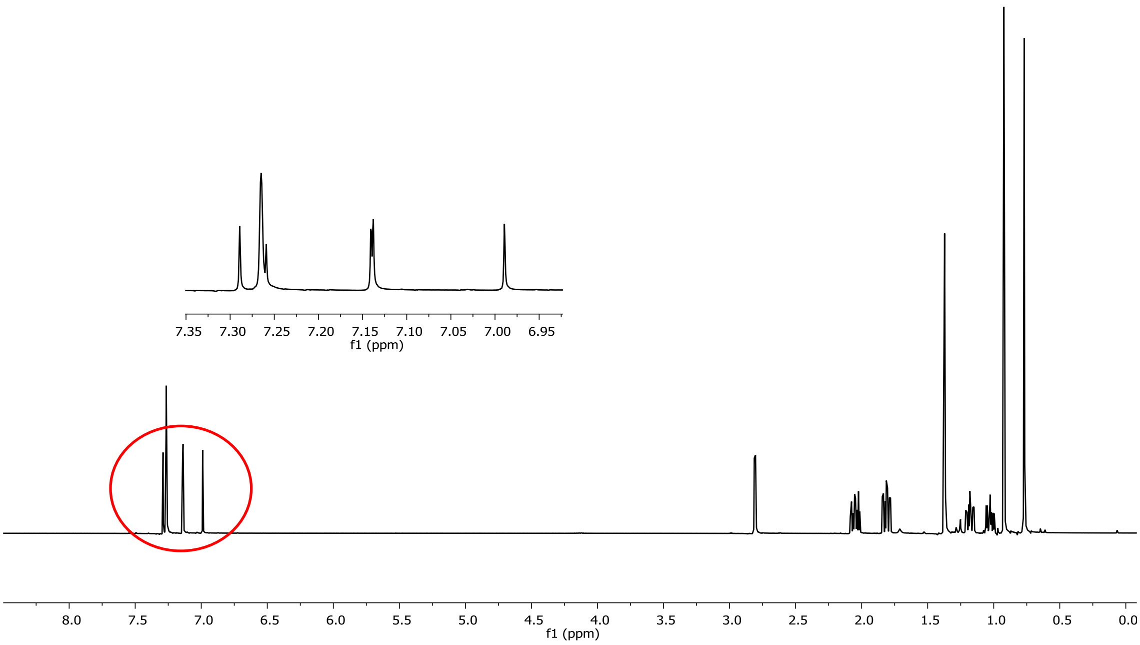

Figure S1: ^{1}H NMR spectrum of **13** in $CDCl_3$ and amplification of circled region.

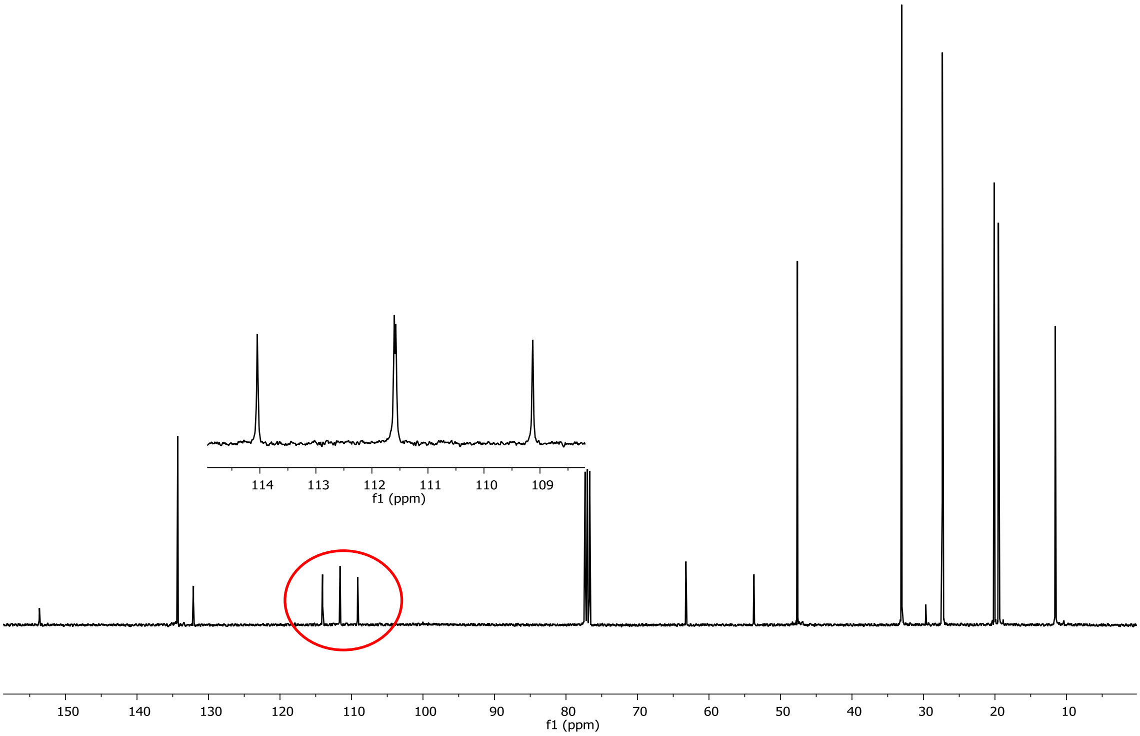

Figure S2: ^{13}C NMR spectrum of **13** in $CDCl_3$ and amplification of circled region.

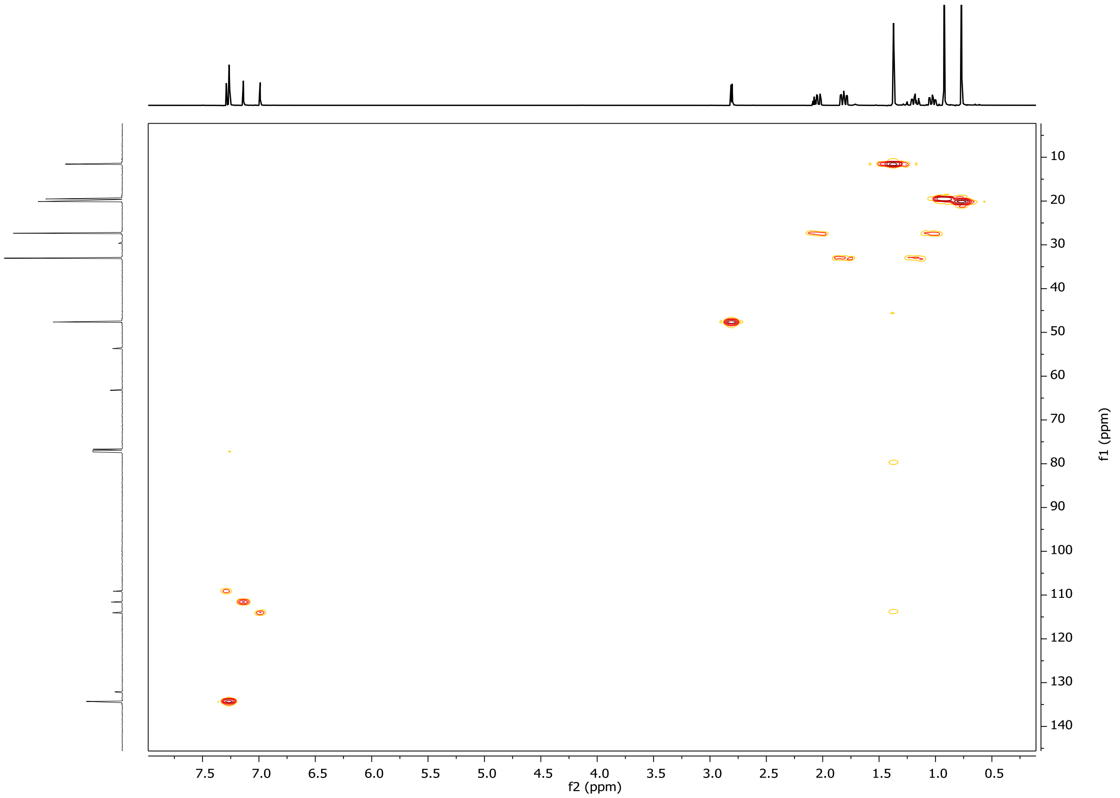

Figure S3: NMR spectrum (^{1}H-^{13}C) gs-HMQC of **13** in $CDCl_3$.

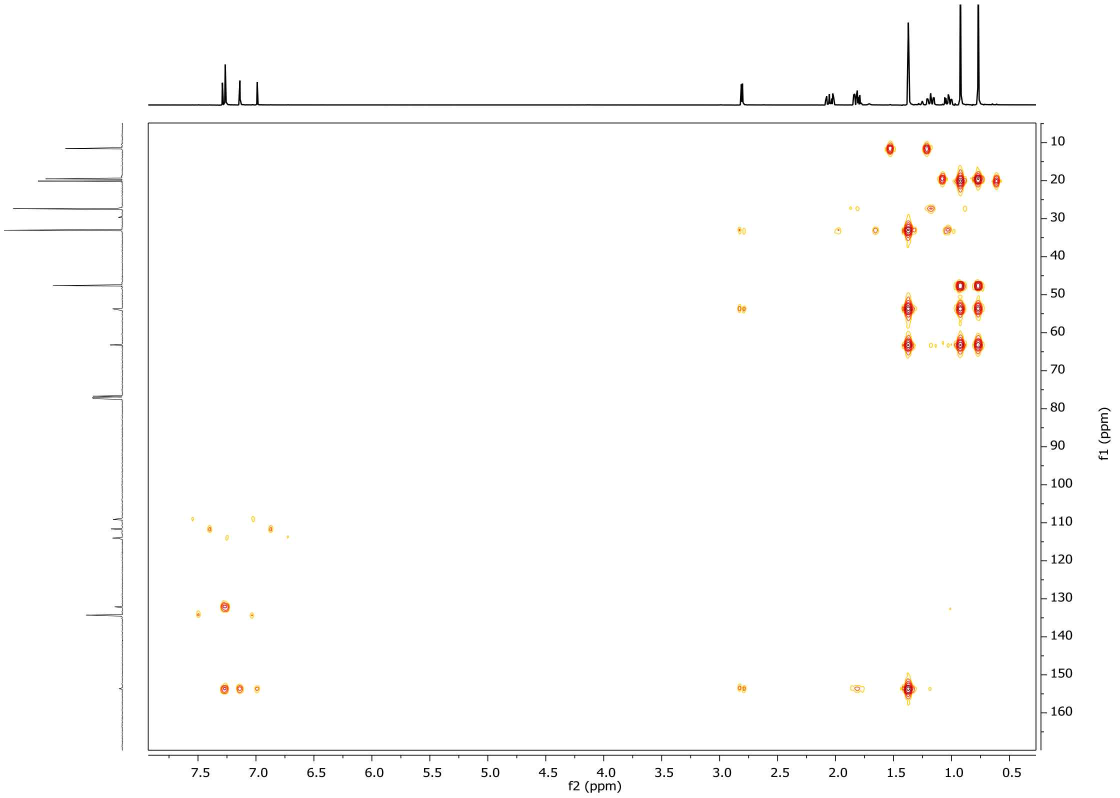

Figure S4: NMR spectrum (^{1}H-^{13}C) gs-HMBC of **13** in $CDCl_3$.

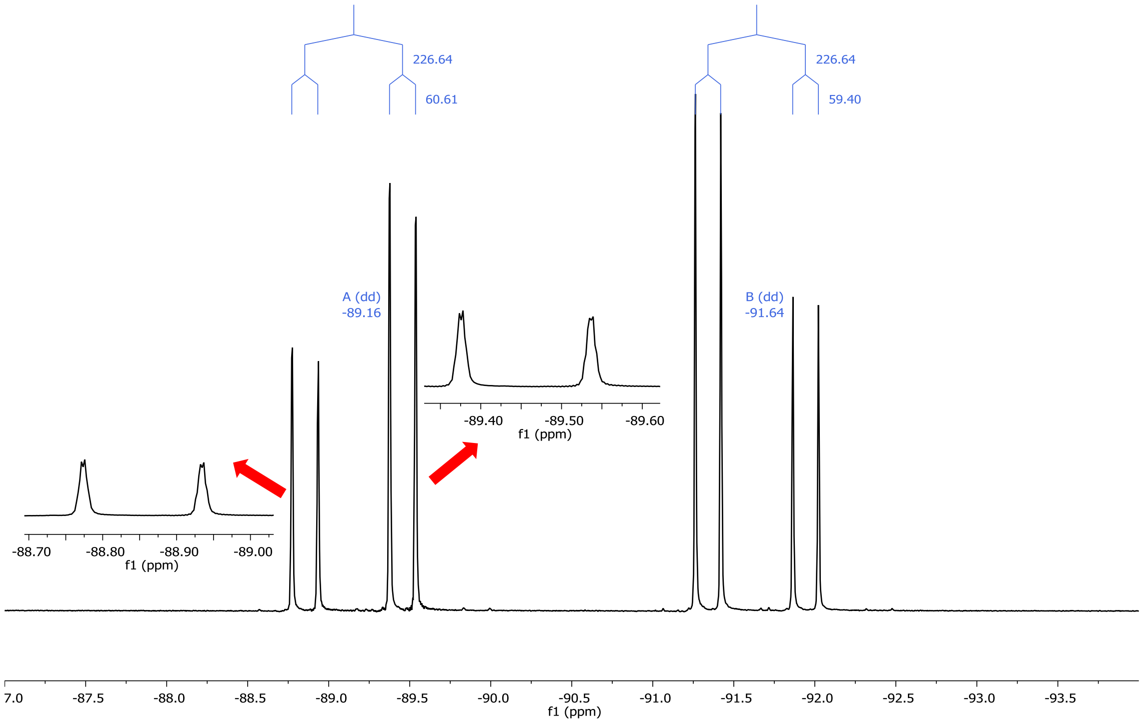

Figure S5: ^{19}F NMR spectrum of **13** in $CDCl_3$.

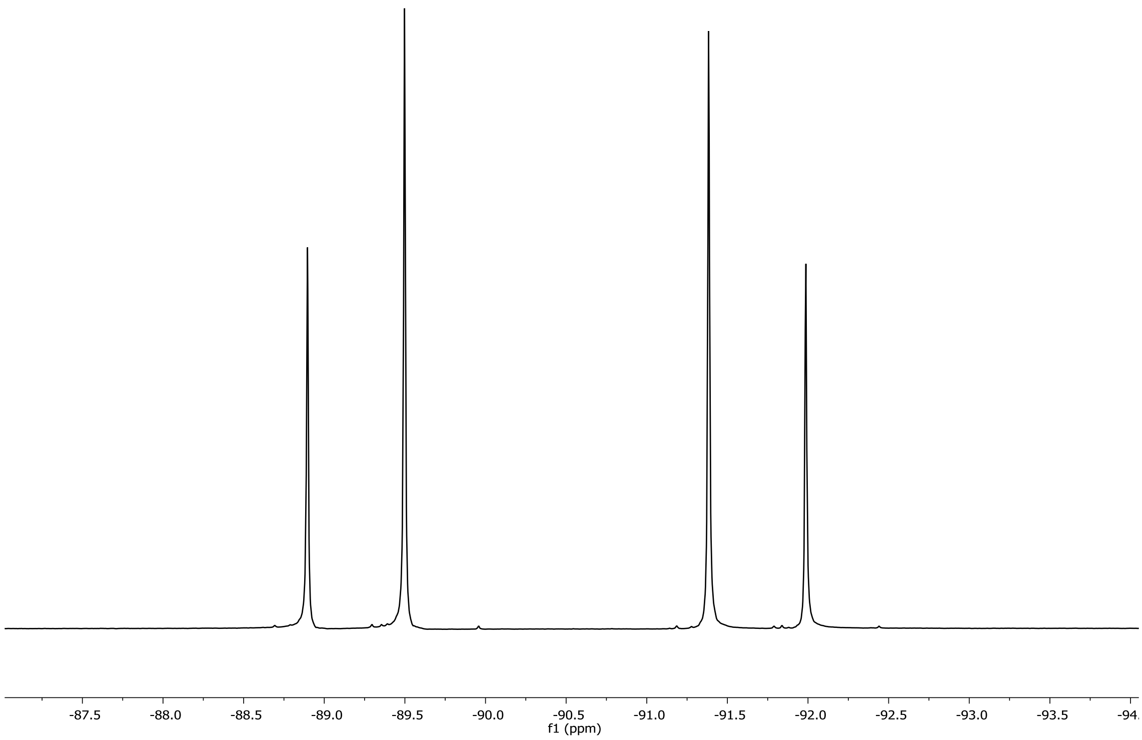

Figure S6: ^{19}F{^{1}H} NMR spectrum of **13** in $CDCl_3$.

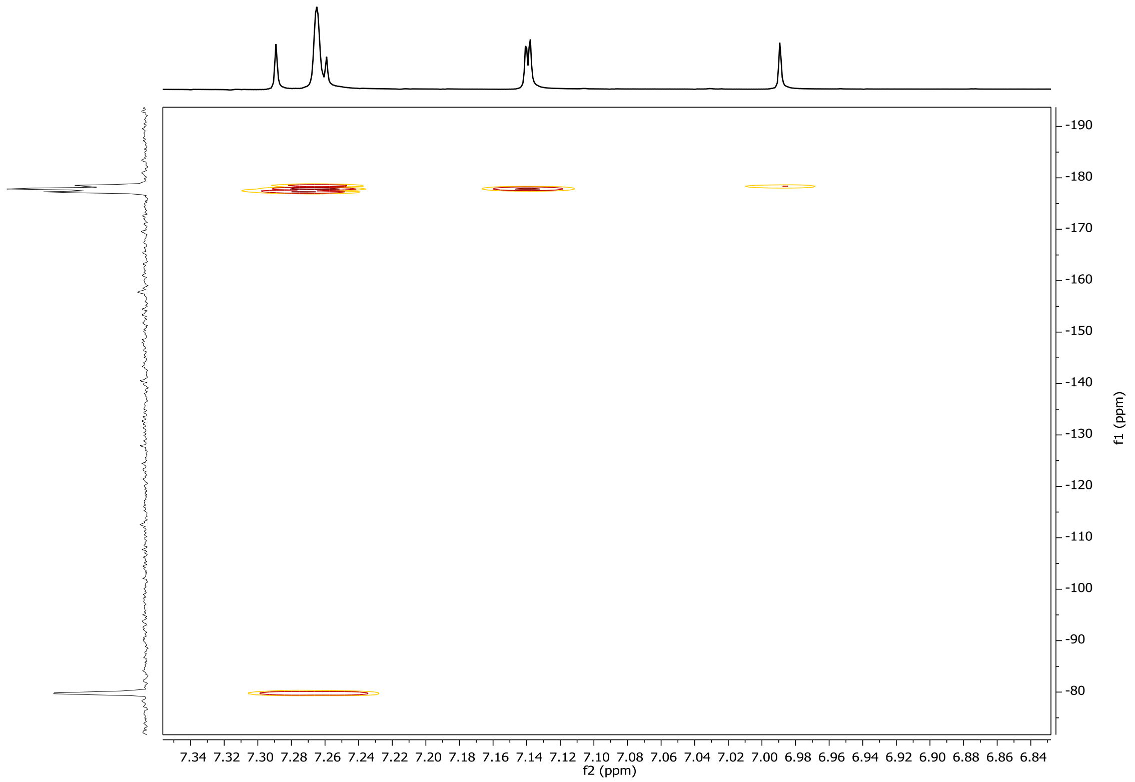

Figure S7: NMR spectrum (^{1}H-^{15}N) gs-HMBC of **13** in $CDCl_3$.

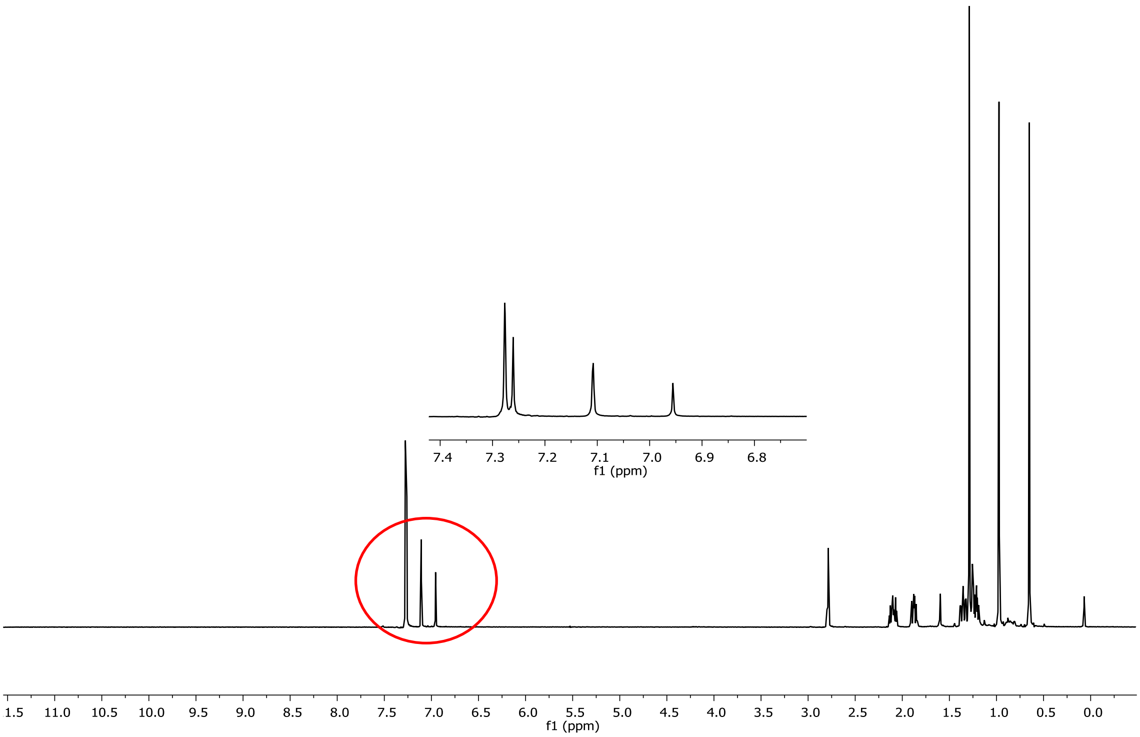

Figure S8: ^{1}H NMR spectrum of **14** in $CDCl_3$ and amplification of circled region.

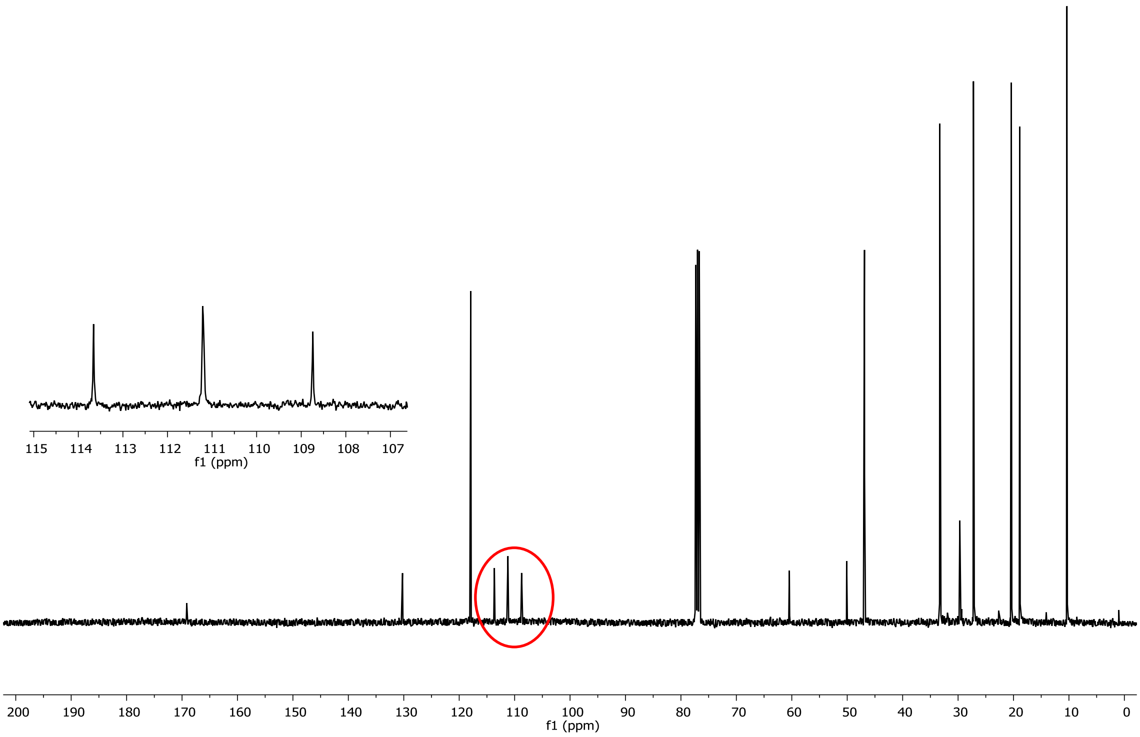

Figure S9: ^{13}C NMR spectrum of **14** in $CDCl_3$ and amplification of circled region.

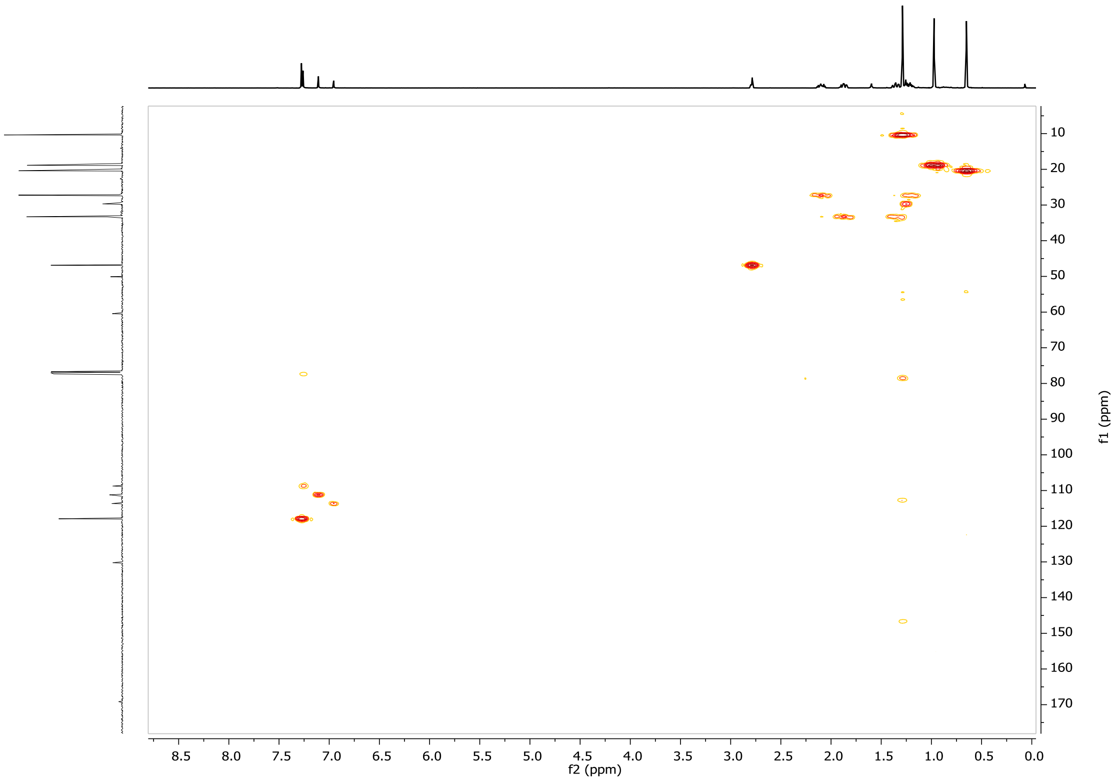

Figure S10: NMR spectrum (^{1}H-^{13}C) gs-HMQC of **14** in $CDCl_3$.

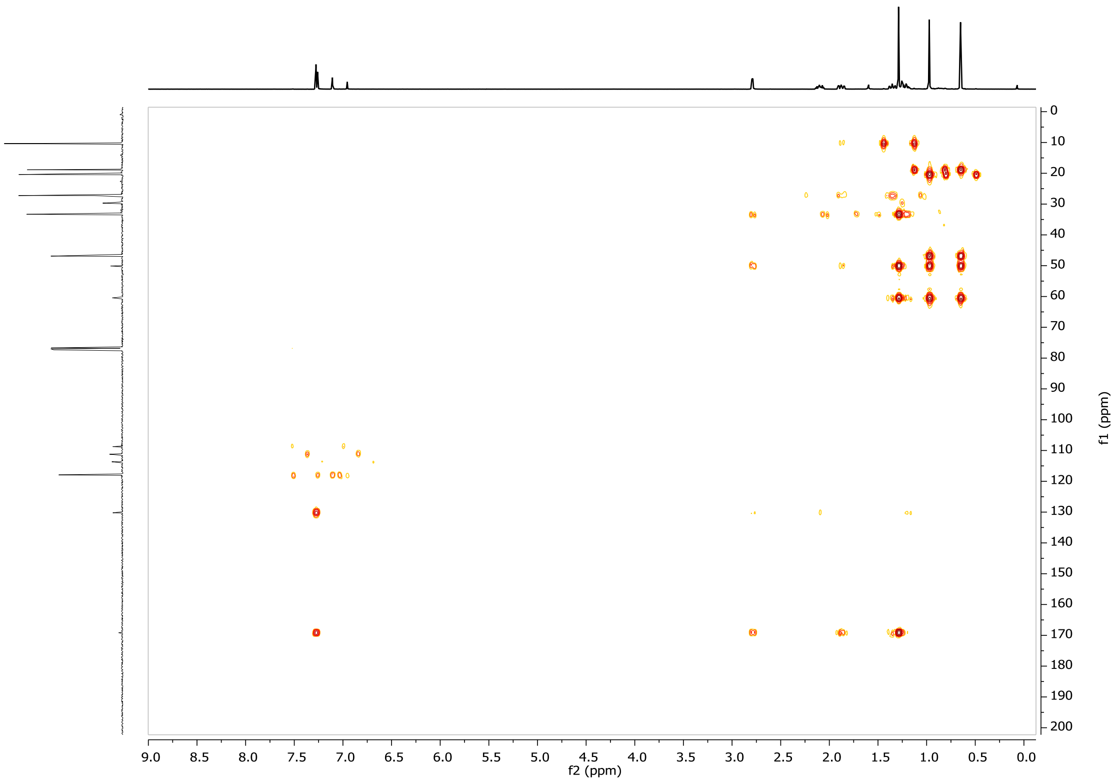

Figure S11: NMR spectrum (^{1}H-^{13}C) gs-HMBC of **14** in $CDCl_3$.

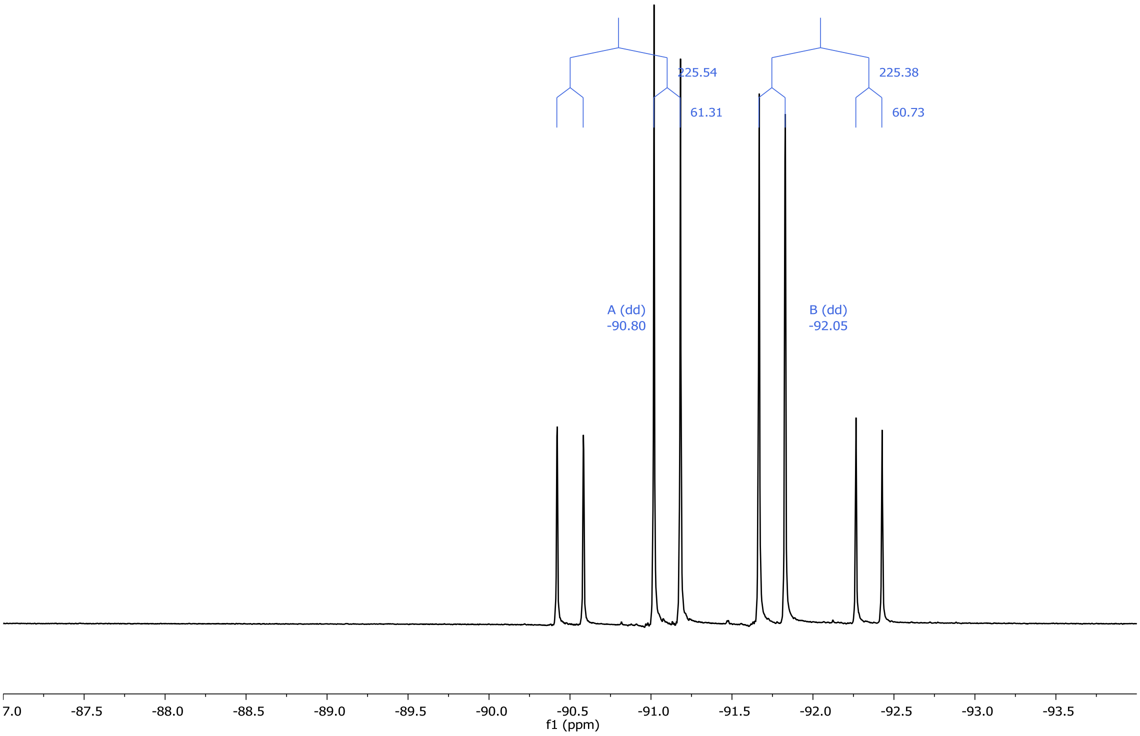

Figure S12: ^{19}F NMR spectrum of **14** in $CDCl_3$.

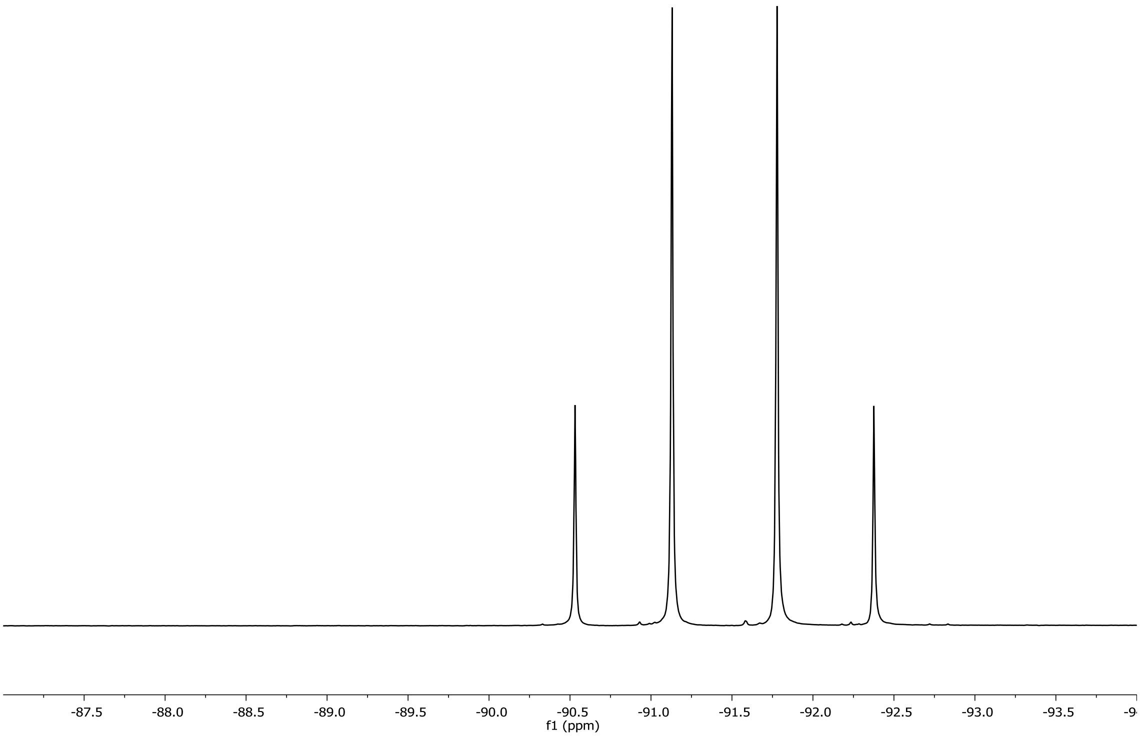

Figure S13: $^{19}F\{^{1}H\}$ NMR spectrum of **14** in $CDCl_3$.

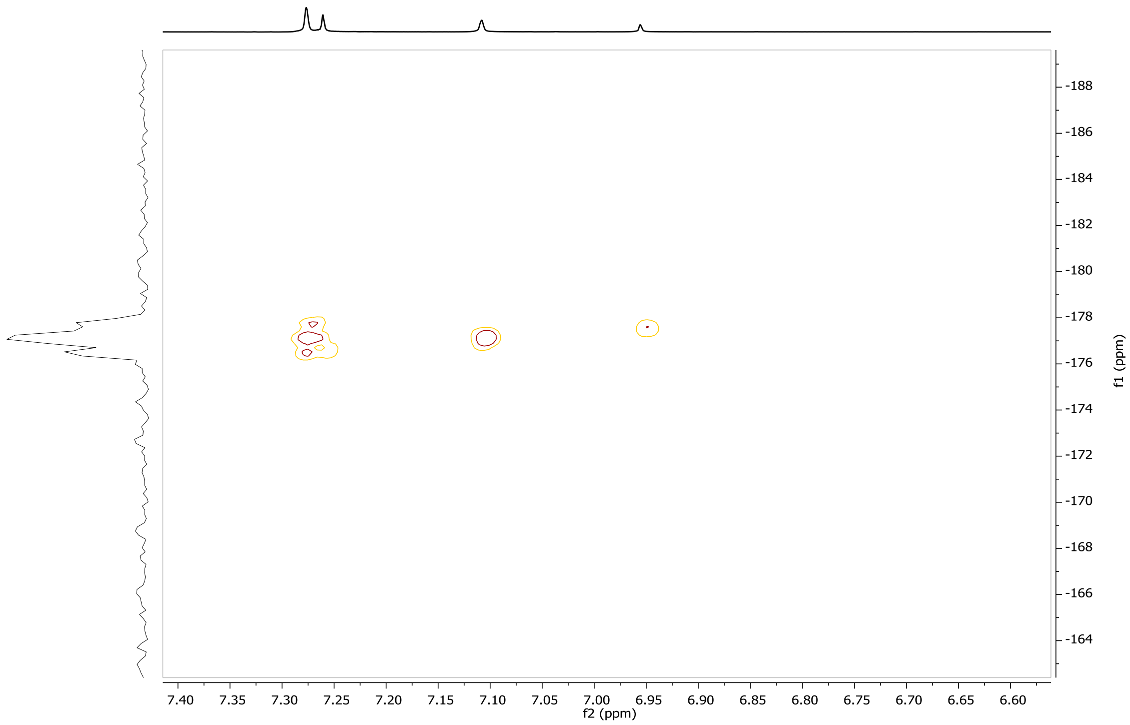

Figure S14: NMR spectrum (^{1}H-^{15}N) gs-HMBC of **14** in $CDCl_3$.

B3LYP/6-311++G(d,p) computational level results in gas phase of the stationary points (minima and TSs).

**Optimized geometry and Electronic energy**	**Chemical shielding and shift (ppm)**
**13** (θ = -3.6°)<br>Electronic Energy= -776.272387310 Hartree<br>NIMAG= 0<br>H,-1.4648424704,1.7387341004,-2.173598583<br>H,0.6143232975,3.5439778359,0.4779123323<br>N,1.8963376686,1.9171782991,0.3069423644<br>C,0.6889934156,2.4758801072,0.3346208904<br>H,-0.2637535688,-0.3251623287,-2.4208879591<br>C,-1.9004184051,0.9283384783,-1.587586778<br>N,1.6668828505,0.577182361,0.102959539<br>H,-2.943387123,0.8275397476,-1.8957243407<br>C,-0.3252283473,1.5051289279,0.1835482123<br>C,-1.1225830994,-0.4129942197,-1.7533656614<br>C,0.3300751159,0.3084939424,0.0424208253<br>C,-1.7900520598,1.2203040768,-0.0543135754<br>H,-2.5090240149,1.9467778512,0.3253441107<br>H,-1.7620764281,-1.2035979286,-2.1533155691<br>C,-0.6602030983,-0.7864972274,-0.2955083467<br>H,0.5710965822,-2.504622286,-0.769182508<br>C,-1.8926944263,-0.2306601786,0.5530289229<br>C,-0.2521856139,-2.2372909569,-0.1022742896<br>H,-3.4715433751,-0.9967351342,-0.8053114873<br>H,-0.7536553523,0.1922590526,2.3935427811<br>C,-3.2249602546,-0.9369451084,0.253920875<br>H,0.0754487757,-2.4325597194,0.9215351788<br>H,-4.0422675951,-0.4076221339,0.7537181576<br>H,-1.0895308084,-2.9031023976,-0.3273405114<br>C,-1.6811556512,-0.2825911096,2.0745414754<br>H,-2.5083143437,0.2211347031,2.5843562495<br>H,-1.669127308,-1.3187974411,2.4250594691<br>H,-3.2138324624,-1.9564300853,0.649687084<br>C,2.7908437596,-0.282475772,0.0514805326<br>H,3.6990148797,0.3105239849,0.1261816241<br>F,2.7957296346,-1.003085912,-1.1135895155<br>F,2.7552868261,-1.2076175289,1.0592325008	Atom Abs. Rel.<br>1H 30.97 0.96<br>2H 24.70 7.04<br>3N -84.24 -72.31<br>4C 44.08 133.25<br>5H 30.66 1.26<br>6C 151.74 29.57<br>7N 23.97 -174.68<br>8H 29.92 1.98<br>9C 44.35 132.99<br>10C 146.11 34.99<br>11C 22.85 153.70<br>12C 128.97 51.50<br>13H 29.21 2.67<br>14H 30.11 1.80<br>15C 122.47 57.76<br>16H 29.95 1.95<br>17C 111.49 68.34<br>18C 169.84 12.15<br>19H 30.94 0.99<br>20H 31.04 0.89<br>21C 162.35 19.36<br>22H 30.47 1.44<br>23H 31.12 0.82<br>24H 30.90 1.03<br>25C 162.17 19.53<br>26H 31.15 0.79<br>27H 31.19 0.75<br>28H 31.01 0.92<br>29C 62.38 115.63<br>30H 24.88 6.87<br>31F 266.19 -93.18<br>32F 262.59 -89.73
**13** (θ = 104.4°)<br>TotalEnergy= -776.263393857 Hartree<br>NIMAG= 1<br>H,-1.4785004245,1.7284508168,-2.1825232561<br>H,0.5909740527,3.5615516717,0.460174193<br>N,1.8889816867,1.9501687693,0.2721054329<br>C,0.6743643548,2.4947130726,0.3125377151<br>H,-0.2737926146,-0.3331817851,-2.430901325<br>C,-1.9089220367,0.9193108702,-1.5912138704<br>N,1.6752522264,0.6156876338,0.0520139113<br>H,-2.9529074001,0.8134073536,-1.8943987612<br>C,-0.3293522253,1.5147727522,0.1702961306	

C,-1.126710387,-0.420358257,-1.7559877983			
C,0.3397505817,0.3247479409,0.0249488118			
C,-1.7919717734,1.2187599815,-0.0600601866			
H,-2.5146335753,1.9416909043,0.3195413393			
H,-1.7670349144,-1.2153053205,-2.1462029916			
C,-0.6505465325,-0.780118496,-0.3000887102			
H,0.5510006339,-2.5234042453,-0.7762785154			
C,-1.8805599201,-0.2276601712,0.5549256951			
C,-0.2499674107,-2.2321251967,-0.0940295133			
H,-3.460065766,-1.0210781699,-0.7869744695			
H,-0.7418380534,0.2314359593,2.3862775255			
C,-3.2107653923,-0.9443813805,0.2705223465			
H,0.0940170018,-2.4161494779,0.928172222			
H,-4.0283837817,-0.4094137619,0.7636185972			
H,-1.1008106276,-2.8920099225,-0.2830653804			
C,-1.6597385673,-0.2672390128,2.0756425626			
H,-2.4944785978,0.222211291,2.5869205384			
H,-1.6253537288,-1.3006378898,2.4337828417			
H,-3.1967715726,-1.9574841584,0.6824436167			
C,2.7797855689,-0.3045955111,0.0549976378			
H,2.8371917911,-0.9088332232,0.9630187719			
F,3.9358159126,0.3573110005,-0.1075487272			
F,2.6279051018,-1.1667247777,-1.0008239639			
**13** (θ = 180.7°)			
TotalEnergy= -776.266405072 Hartree			
NIMAG= 0			
H,-0.1068263604,1.3878197314,-2.060852702	Atom	Abs.	Rel.
H,1.7603989714,-1.9424954794,-2.3111507768	1H	30.84	1.08
N,-0.06585924,-2.7930797263,-1.8042103686	2H	24.51	7.23
C,0.8624524924,-1.8446586631,-1.7184170259	3N	-81.76	-74.66
H,-2.2650321813,0.6834650478,-1.2897874811	4C	42.39	134.88
C,-0.2167263161,1.4885599721,-0.9804858874	5H	30.78	1.14
N,-1.0695907377,-2.3886938014,-0.9655872849	6C	151.07	30.22
H,-0.0208816463,2.5329527666,-0.7277422237	7N	25.30	-175.94
C,0.4871954269,-0.8414600919,-0.7967565356	8H	29.93	1.97
C,-1.6229958093,1.0351434819,-0.4799390626	9C	47.06	130.38
C,-0.7442463276,-1.2212053215,-0.3311177497	10C	145.28	35.80
C,0.7593158965,0.5292569542,-0.2232615222	11C	21.64	154.86
H,1.7968105814,0.863274676,-0.1956872107	12C	129.87	50.64
H,-2.1529580519,1.8480044592,0.0229298025	13H	29.25	2.63
C,-1.3237986783,-0.1249156189,0.5413954776	14H	30.14	1.76
H,-3.3831735282,-0.6928118281,0.9401406487	15C	122.85	57.40
C,0.0470279524,0.3912336016,1.1763094207	16H	30.49	1.42
C,-2.4673788011,-0.4510824241,1.4890622138	17C	111.76	68.08
H,-0.5820895259,2.5091542299,1.3890667221	18C	170.03	11.96
H,0.8592271981,-1.5991333045,1.6659190621	19H	31.08	0.86
C,-0.0802721416,1.71735529,1.9435189889	20H	31.00	0.93
H,-2.22942144,-1.2952744346,2.1421264843	21C	162.44	19.27
H,0.9159382445,2.0831370344,2.2107753716	22H	30.66	1.26
H,-2.6982938424,0.4089336879,2.1230173706	23H	31.09	0.84
C,0.7221899952,-0.6176265073,2.1187110339	24H	30.77	1.15
H,1.7073402941,-0.2469847523,2.4180654853	25C	162.31	19.39

H,0.134908532,-0.7429393182,3.0335911707<br>H,-0.6271921376,1.5689925319,2.8792599593<br>C,-2.2039474066,-3.2186769228,-0.7567714923<br>H,-2.8983620675,-2.7307528112,-0.074400109<br>F,-1.8470992029,-4.4245794584,-0.2287601046<br>F,-2.8420805721,-3.4797696408,-1.929622495	26H 31.19 0.74<br>27H 31.29 0.65<br>28H 31.05 0.88<br>29C 68.48 109.76<br>30H 25.05 6.70<br>31F 262.95 -90.07
**13** (θ = 255.8°)<br>TotalEnergy= -776.262247149 Hartree<br>NIMAG= 1<br>H,-1.4423976701,1.7517621174,-2.1621774931<br>H,0.5896812836,3.5515752677,0.5218439537<br>N,1.8866927413,1.935491375,0.3733000407<br>C,0.6733555449,2.4842089294,0.3780554485<br>H,-0.2280749052,-0.3031000648,-2.4053731088<br>C,-1.8802760904,0.936366165,-1.5850167098<br>N,1.6732581312,0.5958815777,0.1852916164<br>H,-2.9187711666,0.8288864672,-1.9059112529<br>C,-0.3289299327,1.5082192933,0.2072188918<br>C,-1.0934564709,-0.4008407242,-1.7465210509<br>C,0.3396134058,0.3158707795,0.0762523098<br>C,-1.7882490127,1.2199304912,-0.0503334935<br>H,-2.51399285,1.9421634481,0.3246322319<br>H,-1.7242610792,-1.1913365441,-2.1604916836<br>C,-0.6480828817,-0.7812670881,-0.284284401<br>H,0.5628733636,-2.5223418035,-0.7553990994<br>C,-1.8942764734,-0.2326980123,0.5493371169<br>C,-0.2642708936,-2.2398019011,-0.0986471377<br>H,-3.4493658648,-1.0057821755,-0.8331948304<br>H,-0.7836635556,0.1933762503,2.4048032542<br>C,-3.2202822292,-0.9423394261,0.229831974<br>H,0.0457064043,-2.4512184767,0.9264429718<br>H,-4.0462583164,-0.4114441179,0.7133886801<br>H,-1.1098986912,-2.8875211065,-0.3448678772<br>C,-1.7021700754,-0.2896507415,2.0730348456<br>H,-2.5406925211,0.2039052168,2.5741043651<br>H,-1.6849395124,-1.3271881492,2.4201668438<br>H,-3.2166701281,-1.9602594591,0.6299257705<br>C,2.7906233743,-0.2957150796,0.0232167889<br>H,2.940076357,-0.6114988103,-1.012072177<br>F,2.5659268193,-1.4229517199,0.7665870015<br>F,3.9158809454,0.2851456816,0.4689763402	

**14** (θ = 11.1°)<br>TotalEnergy= -776.277457829 Hartree<br>NIMAG= 0<br>H,0.3148010234,1.8212548542,-2.0896407105<br>H,2.2002069469,-1.5920802216,-2.3638765628<br>N,0.2691753301,-2.3538489982,-1.8157197801<br>C,1.3075388769,-1.4348037757,-1.7805257635<br>H,-1.8561905583,1.0949476338,-1.3721542741<br>C,0.1939155815,1.8729717216,-1.0067361736	 <br>Atom Abs. Rel.<br>1H 30.73 1.19<br>2H 24.57 7.17<br>3N 21.29 -172.14<br>4C 60.68 117.26<br>5H 30.55 1.37<br>6C 150.69 30.58

N,-0.7860508495,-2.0023553797,-1.0020572638<br>H,0.3960527114,2.9028254103,-0.7052159183<br>C,0.8984134408,-0.4577057763,-0.9203501204<br>C,-1.2214910323,1.4063937949,-0.5407002096<br>C,-0.3885678642,-0.8639062115,-0.4821513134<br>C,1.1595872615,0.8814625735,-0.2825049428<br>H,2.1962421201,1.213906565,-0.2235460898<br>H,-1.7510653655,2.2006998691,-0.009389407<br>C,-0.9446886174,0.2025369384,0.4285066142<br>H,-2.9708256568,-0.4831111354,0.735438152<br>C,0.4230751283,0.6733239921,1.100828406<br>C,-2.1039610999,-0.1825324462,1.3303773462<br>H,-0.1839410145,2.7827938483,1.4165945408<br>H,1.1994849717,-1.3542365167,1.484009122<br>C,0.3044521901,1.9582391693,1.9348787575<br>H,-1.8443979966,-1.023801051,1.9781110969<br>H,1.3001562529,2.299956301,2.2345613401<br>H,-2.4070655658,0.6575400759,1.9615191576<br>C,1.0799732458,-0.395001091,1.9885409558<br>H,2.0712042414,-0.0600735521,2.3099885067<br>H,0.485781952,-0.5621528214,2.8917280683<br>H,-0.2587448584,1.7655038993,2.8525065172<br>C,0.2431612119,-3.6164739075,-2.4470629291<br>H,-0.743375117,-4.0594915301,-2.3332943634<br>F,1.1868242408,-4.4651159677,-1.9216223015<br>F,0.5605297687,-3.4966921047,-3.7732668776	7N -63.30 -92.12<br>8H 29.89 2.01<br>9C 45.04 132.33<br>10C 145.08 35.99<br>11C 8.55 167.47<br>12C 130.25 50.27<br>13H 29.26 2.62<br>14H 30.09 1.81<br>15C 126.23 54.14<br>16H 30.21 1.69<br>17C 115.04 64.92<br>18C 171.39 10.65<br>19H 30.96 0.97<br>20H 31.35 0.59<br>21C 163.05 18.68<br>22H 30.68 1.24<br>23H 31.21 0.72<br>24H 30.95 0.98<br>25C 161.57 20.11<br>26H 31.25 0.69<br>27H 31.18 0.76<br>28H 31.08 0.85<br>29C 62.88 115.15<br>30H 24.87 6.87<br>31F 258.43 -85.73<br>32F 270.59 -97.40
**14** (θ = 114.9°)<br>TotalEnergy= -776.268491134 Hartree<br>NIMAG= 1<br>H,0.2540974946,1.8003445468,-2.1034831219<br>H,2.1886620803,-1.6234019617,-2.3334611793<br>N,0.2737914987,-2.398588142,-1.7399532497<br>C,1.2916458289,-1.4567620084,-1.7585999341<br>H,-1.8981879003,1.0664787105,-1.3380633337<br>C,0.1525006537,1.8638950114,-1.0191126891<br>N,-0.7800681072,-2.032218728,-0.9398913714<br>H,0.3506479902,2.8992173999,-0.7338100399<br>C,0.8817078646,-0.4590342362,-0.9207059573<br>C,-1.2502419054,1.3905446335,-0.5216777032<br>C,-0.3907749796,-0.8727335808,-0.4575969374<br>C,1.1406708804,0.8892210763,-0.3014304154<br>H,2.1745371613,1.2333786448,-0.2627867364<br>H,-1.7757152891,2.1862009422,0.0119188595<br>C,-0.9443810946,0.1973827113,0.4516131839<br>H,-2.955484696,-0.5111955197,0.7969591677<br>C,0.4305396843,0.6875944909,1.0964084995<br>C,-2.083850632,-0.1933281234,1.3756383805<br>H,-0.1780940971,2.797383363,1.4015214342<br>H,1.2272611794,-1.3314486913,1.4850703734<br>C,0.3140624574,1.9770807564,1.9229012676<br>H,-1.8033129648,-1.0260115877,2.0254155745<br>H,1.3104632322,2.3222096531,2.2165299186	

H,-2.3878637893,0.6483129441,2.0043292384			
C,1.1128782584,-0.3673870071,1.9814585412			
H,2.1085699927,-0.023000677,2.2784414511			
H,0.5389971862,-0.5282316183,2.8987188431			
H,-0.2451540492,1.7881624739,2.843853124			
C,0.2626905684,-3.6144131311,-2.4978484851			
H,0.2651060012,-3.445633595,-3.5774545542			
F,-0.8056053862,-4.3579680033,-2.1692464363			
F,1.3970749778,-4.3365822172,-2.2085806331			
**14** (θ = 179.3°)			
TotalEnergy= -776.272975736 Hartree			
NIMAG= 0	Atom	Abs.	Rel.
H,-1.2158240623,2.5905506276,-1.136599604	1H	30.84	1.08
H,1.7205186828,2.0320845775,1.3805378237	2H	25.06	6.69
N,2.0143406325,0.2718054959,0.1774778781	3N	23.50	-174.23
C,1.2501721037,1.2461128496,0.8108121535	4C	58.64	119.23
H,-1.0498615102,0.6139410089,-2.4863440491	5H	30.59	1.33
C,-1.8903454934,1.8000932056,-0.8045041205	6C	150.96	30.33
N,1.2516288332,-0.6509856414,-0.4935517801	7N	-60.79	-94.49
H,-2.9022605966,2.2107370137,-0.8127924695	8H	29.82	2.08
C,-0.046900043,0.9334740653,0.5305865116	9C	49.30	128.22
C,-1.7858120886,0.515461389,-1.6864622045	10C	145.60	35.49
C,0.0260718245,-0.2350470118,-0.2718188833	11C	5.68	170.23
C,-1.5066747079,1.2926170525,0.6226972361	12C	129.57	50.92
H,-1.8106314053,1.948829192,1.4386516868	13H	29.31	2.57
H,-2.742114912,0.2717037264,-2.1552493754	14H	30.05	1.85
C,-1.3721410282,-0.6215942013,-0.6861281052	15C	125.87	54.48
H,-0.9856971702,-2.2204294749,-2.0861284372	16H	30.18	1.73
C,-2.1437669747,-0.1532995117,0.6293895989	17C	115.27	64.70
C,-1.5835415835,-2.0382639661,-1.1889780374	18C	171.35	10.69
H,-4.0448443249,0.3814589185,-0.3773643569	19H	30.81	1.11
H,-0.7153233058,-1.0447926518,2.0540257621	20H	31.39	0.55
C,-3.674085857,-0.1552472931,0.4951039216	21C	162.76	18.96
H,-1.2821419141,-2.7760354222,-0.4411379584	22H	30.70	1.23
H,-4.1262502623,0.3015251818,1.3811422437	23H	31.09	0.85
H,-2.6324254202,-2.2136088284,-1.4439652253	24H	31.02	0.91
C,-1.7894978382,-0.9722088388,1.8807660861	25C	161.58	20.09
H,-2.2425743785,-0.5193934234,2.7683125095	26H	31.24	0.70
H,-2.1845121703,-1.9888306252,1.7977729182	27H	31.17	0.77
H,-4.0482608106,-1.1812893954,0.4356344319	28H	30.88	1.05
C,3.4190087855,0.1300868368,0.2646395096	29C	66.77	111.40
H,3.8380059472,0.9677356064,0.8236985183	30H	25.29	6.47
F,3.9927952136,0.0900160659,-0.9710638762	31F	265.83	-92.83
F,3.773168835,-1.0327275282,0.8888146934	32F	261.48	-88.66

**14** (θ = 242.9°)<br>TotalEnergy= -776.268646637 Hartree<br>NIMAG= 1<br>H,0.3548688818,1.8022442739,-2.0876603034<br>H,2.1927155571,-1.6311087115,-2.3306551722<br>N,0.2477673648,-2.3695054636,-1.7898837953<br>C,1.2997915761,-1.466370129,-1.7486189809<br>H,-1.8335332827,1.1126789955,-1.3873449999<br>C,0.2237217867,1.8632317012,-1.0064812745<br>N,-0.8080834167,-1.9977213325,-0.9946878579<br>H,0.4370026477,2.8923896786,-0.7097800008<br>C,0.8986161782,-0.4754025328,-0.8988545941<br>C,-1.2021030667,1.4185517931,-0.5514341867<br>C,-0.396927988,-0.8617899706,-0.4759436883<br>C,1.1694011332,0.863855215,-0.2658315441<br>H,2.2090805027,1.1849356978,-0.1957379822<br>H,-1.7251071777,2.2224764214,-0.0280980827<br>C,-0.9508513973,0.2152345584,0.4250840555<br>H,-2.9827847789,-0.4596811139,0.7065650969<br>C,0.4150750153,0.6724377415,1.1101402651<br>C,-2.1262416595,-0.152806048,1.3130163429<br>H,-0.1618510717,2.7922443269,1.4071334056<br>H,1.1561952798,-1.3637265622,1.5139447224<br>C,0.3051618704,1.9627415428,1.9367934747<br>H,-1.8813557536,-0.9864782856,1.9759213536<br>H,1.3018671665,2.2898399671,2.2496855245<br>H,-2.438651164,0.6965633184,1.9272020371<br>C,1.0476877882,-0.3994770149,2.01131358<br>H,2.0407877118,-0.0776287368,2.3405813287<br>H,0.4421628619,-0.5518452312,2.9096236972<br>H,-0.2743350572,1.7828858294,2.8468687166<br>C,0.2719700701,-3.6234563793,-2.4815056998<br>H,1.087254443,-4.2734734494,-2.1546543504<br>F,0.4624747413,-3.3959139557,-3.8249368179<br>F,-0.8972664225,-4.2644020241,-2.3267169897	

PCM/($CHCl_3$)//B3LYP/6-311++G(d,p) computational level results of the stationary points (minima and TSs).

Optimized geometry and Electronic energy	Chemical shielding and shift (ppm)
**13** (θ = -4.0°)<br>Electronic Energy= -776.276685713 Hartree<br>NIMAG= 0<br>H,-1.4671383842,1.7397196637,-2.1740150084<br>H,0.604795361,3.5455346367,0.4770841192<br>N,1.8954164235,1.9215802394,0.3076344288<br>C,0.6846316397,2.4775147518,0.334394462<br>H,-0.2640845745,-0.3229077603,-2.4229720414<br>C,-1.9008665622,0.9286406658,-1.5874806313<br>N,1.6686958144,0.5788175446,0.1020880265<br>H,-2.9440062582,0.8260412485,-1.8932547041<br>C,-0.3256210667,1.5051808895,0.181757324<br>C,-1.1219819039,-0.4117114797,-1.7542723675<br>C,0.3318418087,0.3090605802,0.0403580803<br>C,-1.7903251172,1.2203411199,-0.0543482433<br>H,-2.5081492215,1.9467684571,0.3263178809<br>H,-1.7614142335,-1.2029253403,-2.1522488205<br>C,-0.658798711,-0.786505257,-0.2966741583<br>H,0.5680892938,-2.5077619662,-0.7722750966<br>C,-1.8911259975,-0.2306862796,0.5527330916<br>C,-0.2535334202,-2.2380435961,-0.1041706331<br>H,-3.4694209208,-0.9977833301,-0.8048396596<br>H,-0.749659828,0.1894124002,2.3933346967<br>C,-3.2231926395,-0.9373228661,0.2543223311<br>H,0.071121884,-2.4351887928,0.9202909264<br>H,-4.0390533752,-0.4058671769,0.7539657794<br>H,-1.0931155441,-2.9003662434,-0.3299374226<br>C,-1.6787076662,-0.2830892565,2.07423691<br>H,-2.5048441975,0.222954116,2.5831243469<br>H,-1.6687721906,-1.3195656678,2.423796088<br>H,-3.2112786293,-1.9562847257,0.6511169841<br>C,2.7919559305,-0.2804683922,0.0536459769<br>H,3.7046985381,0.3046035547,0.1246393495<br>F,2.7931642206,-1.0106989217,-1.1071674419<br>F,2.7538765276,-1.2043268154,1.0668494266	 <br>Atom Abs. Rel.<br>1H 30.97 0.96<br>2H 24.59 7.15<br>3N -77.27 -78.90<br>4C 42.92 134.36<br>5H 30.68 1.24<br>6C 151.90 29.42<br>7N 24.66 -175.33<br>8H 29.85 2.05<br>9C 43.45 133.86<br>10C 146.20 34.91<br>11C 21.27 155.22<br>12C 129.10 51.38<br>13H 29.11 2.77<br>14H 30.02 1.88<br>15C 122.00 58.21<br>16H 30.02 1.88<br>17C 111.07 68.74<br>18C 169.95 12.04<br>19H 30.89 1.04<br>20H 31.14 0.79<br>21C 162.68 19.04<br>22H 30.49 1.42<br>23H 31.07 0.86<br>24H 30.80 1.12<br>25C 162.44 19.27<br>26H 31.09 0.84<br>27H 31.15 0.78<br>28H 30.98 0.95<br>29C 62.05 115.94<br>30H 24.78 6.96<br>31F 267.12 -94.07<br>32F 263.12 -90.23
**13** (θ = 96.0°)<br>Electronic Energy= -776.269387625 Hartree<br>NIMAG= 1<br>H,-1.4920089009,1.7352195558,-2.1853061422<br>H,0.5806467506,3.5562269229,0.4733079093<br>N,1.8880894321,1.947990504,0.2800360552<br>C,0.670001969,2.4906379658,0.3184921434<br>H,-0.2885179417,-0.3262353053,-2.4476508509<br>C,-1.9178079945,0.9240098065,-1.5932314968<br>N,1.6783234429,0.6134268234,0.0400951977<br>H,-2.9637403589,0.8180333757,-1.8883962941<br>C,-0.3279612369,1.5109835653,0.1603497029	

C,-1.1366098599,-0.415016615,-1.7666031719			
C,0.3429484268,0.3211479072,0.0073334417			
C,-1.7915260724,1.2182847052,-0.0623774821			
H,-2.5092477477,1.9415568216,0.324763968			
H,-1.7797737825,-1.208856282,-2.1532659928			
C,-0.6521422094,-0.7823195576,-0.315066581			
H,0.5164351125,-2.5403334881,-0.8191600113			
C,-1.8764227605,-0.2293379709,0.5491531331			
C,-0.2618215485,-2.2379396613,-0.1164383341			
H,-3.4630167696,-1.0199489132,-0.7852158713			
H,-0.7216500573,0.2166003801,2.3748099383			
C,-3.2093380965,-0.9431012546,0.2711412434			
H,0.1076038201,-2.4243969578,0.8959453955			
H,-4.0220226076,-0.4039269917,0.7676498075			
H,-1.1268504286,-2.88513334,-0.2823841841			
C,-1.6457353961,-0.2738428414,2.0683483201			
H,-2.4740825696,0.2207349988,2.5849716414			
H,-1.6154057082,-1.3084496326,2.4228098442			
H,-3.1953456112,-1.9555669295,0.6840659966			
C,2.7909533739,-0.2966483236,0.0947261096			
H,2.9008925489,-0.803351091,1.0541131828			
F,3.9342537482,0.3645189485,-0.1862334854			
F,2.6128126439,-1.2514478649,-0.8689407123			
**13** (θ = 180.5 °)			
Electronic Energy= -776.273758182 Hartree			
NIMAG= 0	Atom	Abs.	Rel.
H,-0.1082715385,1.3933774433,-2.0616038128	1H	30.86	1.07
H,1.7639732506,-1.9411879274,-2.3029498347	2H	24.42	7.32
N,-0.0626738255,-2.7990504507,-1.7951183259	3N	-68.92	-86.80
C,0.8641407087,-1.8450962944,-1.7123569877	4C	40.96	136.25
H,-2.2674093249,0.6863923584,-1.2890325184	5H	30.75	1.18
C,-0.217898193,1.4910284893,-0.9807694383	6C	151.28	30.02
N,-1.0699986056,-2.3908418867,-0.956149629	7N	26.57	-177.13
H,-0.0207999858,2.5338956816,-0.7242949164	8H	29.86	2.03
C,0.4840278795,-0.8384513371,-0.799639777	9C	46.14	131.27
C,-1.6237989962,1.0372651638,-0.4802890677	10C	145.56	35.53
C,-0.7481960965,-1.2172224616,-0.3328655925	11C	18.83	157.57
C,0.7587156951,0.5313098709,-0.2254143087	12C	129.92	50.59
H,1.7965802256,0.8628133339,-0.1993531278	13H	29.16	2.71
H,-2.1523964931,1.8483677392,0.025811008	14H	30.05	1.85
C,-1.3260657359,-0.1226934286,0.5420516256	15C	122.07	58.15
H,-3.3844773266,-0.6912690126,0.9399626726	16H	30.41	1.50
C,0.0472590664,0.3914073421,1.174666086	17C	111.14	68.67
C,-2.4697090967,-0.4481357925,1.4893127287	18C	170.68	11.34
H,-0.5810973618,2.5084066427,1.3905257338	19H	31.02	0.91
H,0.857324116,-1.6012840968,1.662137166	20H	31.16	0.77
C,-0.0780148936,1.7164554021,1.9434036812	21C	162.76	18.96
H,-2.2314818393,-1.2899720756,2.1450623731	22H	30.64	1.28
H,0.9195688547,2.0804085025,2.2077840526	23H	31.07	0.86
H,-2.6994548514,0.4144979752,2.1196891747	24H	30.73	1.19
C,0.7224620862,-0.6191828233,2.1152462611	25C	162.56	19.15
H,1.7083355789,-0.2491252731,2.4126336033	26H	31.15	0.78

H,0.1348444138,-0.7444711876,3.0296557365	27H 31.22 0.72
H,-0.6238584367,1.5657222953,2.8791600631	28H 31.00 0.93
C,-2.2037875137,-3.2167881667,-0.7524277689	29C 67.50 110.69
H,-2.9045073405,-2.7409431065,-0.0707011823	30H 24.86 6.89
F,-1.8438070855,-4.4343815733,-0.2394775094	31F 264.93 -91.97
F,-2.8349477646,-3.4801079854,-1.9353189888	32F 272.61 -99.34
**13** (θ = 261.7 °)	
Electronic Energy= -776.268496900 Hartree	
NIMAG= 1	
H,-1.4407805367,1.7462579578,-2.1644190266	
H,0.5820081841,3.5507451876,0.5101312471	
N,1.8864792052,1.9346806649,0.3723176344	
C,0.6699029327,2.4826406143,0.3725289496	
H,-0.221824202,-0.3093588318,-2.398673573	
C,-1.8775248914,0.9315860275,-1.5851588239	
N,1.6739654191,0.5907624266,0.1956552497	
H,-2.915953352,0.8219828134,-1.9045372005	
C,-0.3284397579,1.5054017204,0.2055262746	
C,-1.089444711,-0.4048848173,-1.7428577689	
C,0.3412685229,0.3112442878,0.0802888271	
C,-1.7877000575,1.2186272149,-0.0512781257	
H,-2.5114984181,1.9432429563,0.3217788377	
H,-1.7192604291,-1.1966221066,-2.1549962424	
C,-0.6487602505,-0.785472312,-0.2787839155	
H,0.5479912993,-2.5329827484,-0.7550409166	
C,-1.8947591505,-0.2319238054,0.5521167814	
C,-0.2733996648,-2.2460680265,-0.0937639911	
H,-3.4462274895,-1.0099260315,-0.8301583914	
H,-0.7847521578,0.1971951064,2.4080111672	
C,-3.2208078842,-0.9410903222,0.2332190711	
H,0.0364011632,-2.4602488077,0.9309987747	
H,-4.0461372345,-0.4049525625,0.7118960005	
H,-1.1263272724,-2.8852469399,-0.3360968186	
C,-1.7045296358,-0.2843088477,2.0762908908	
H,-2.5425927158,0.2137199008,2.573502448	
H,-1.6900646952,-1.3208890189,2.4260636628	
H,-3.2183854681,-1.9567264573,0.6386900604	
C,2.7988620724,-0.2867245776,0.0084882132	
H,2.9953148252,-0.5286469802,-1.0368204413	
F,2.5556516086,-1.456426926,0.6729837767	
F,3.9060327923,0.2702409006,0.5442174989	

**14** (θ = 11.2 °)	
Electronic Energy= -776.282012844 Hartree	
NIMAG= 0	Atom Abs. Rel.
H,0.3136468322,1.8223250426,-2.0899951395	1H 30.73 1.19
H,2.1999523801,-1.5865052276,-2.3628502789	2H 24.44 7.29
N,0.2676625514,-2.3555916287,-1.8183566215	3N 23.34 -174.08
C,1.3053098752,-1.4334687964,-1.7811462691	4C 59.74 118.17
H,-1.8582965763,1.0973455079,-1.3723915406	5H 30.57 1.35
C,0.1929672819,1.8727250441,-1.0069637304	6C 150.89 30.40

N,-0.7895460017,-2.0034518051,-1.0020135785<br>H,0.3960667333,2.901420317,-0.7033029893<br>C,0.8944976716,-0.4576979878,-0.9215088705<br>C,-1.2224952305,1.4071179039,-0.5410163861<br>C,-0.3926745106,-0.8638873601,-0.4822518939<br>C,1.1582404589,0.8805814768,-0.2834496314<br>H,2.1953089369,1.210513479,-0.2260811399<br>H,-1.7504334217,2.2008750411,-0.0079258568<br>C,-0.9470853624,0.2031377375,0.4290287411<br>H,-2.978322347,-0.468987552,0.7415387214<br>C,0.4224022423,0.6720949406,1.0999634069<br>C,-2.1060823922,-0.1772759803,1.3334069222<br>H,-0.183846557,2.781033189,1.4179768865<br>H,1.1992156718,-1.3564359159,1.4817562455<br>C,0.3057090292,1.9565125962,1.9349503295<br>H,-1.8493668224,-1.0197762527,1.9808973493<br>H,1.3029936682,2.2966168967,2.230722254<br>H,-2.4003131432,0.6643296022,1.9662882038<br>C,1.0787404328,-0.3973231313,1.9870269581<br>H,2.0694884971,-0.0614671985,2.3084515449<br>H,0.4833549523,-0.5653069573,2.8891942609<br>H,-0.2551566036,1.7625842424,2.8536589838<br>C,0.2456146641,-3.6166003109,-2.4461643914<br>H,-0.735516324,-4.0707019394,-2.3373948101<br>F,1.198108592,-4.4635457693,-1.9201398112<br>F,0.5700657214,-3.5002150439,-3.7741342891	7N -57.80 -97.32<br>8H 29.82 2.07<br>9C 43.71 133.60<br>10C 145.20 35.87<br>11C 6.68 169.27<br>12C 130.44 50.08<br>13H 29.16 2.71<br>14H 30.01 1.89<br>15C 125.94 54.42<br>16H 30.27 1.64<br>17C 114.77 65.18<br>18C 171.63 10.42<br>19H 30.91 1.02<br>20H 31.45 0.49<br>21C 163.39 18.35<br>22H 30.69 1.23<br>23H 31.17 0.77<br>24H 30.86 1.06<br>25C 161.82 19.87<br>26H 31.19 0.74<br>27H 31.14 0.80<br>28H 31.06 0.88<br>29C 62.31 115.69<br>30H 24.76 6.98<br>31F 259.07 -86.35<br>32F 271.21 -97.99
**14** (θ = 110.0 °)<br>Electronic Energy= -776.274458669 Hartree<br>NIMAG= 1<br>H,0.2616859748,1.7866807943,-2.1055231055<br>H,2.1965885129,-1.6278054648,-2.3217746289<br>N,0.2892089618,-2.4141049699,-1.7218443286<br>C,1.3010173547,-1.4663139532,-1.7433945792<br>H,-1.8932100132,1.0555278405,-1.3436759259<br>C,0.1566775049,1.8544126701,-1.0217069207<br>N,-0.7739306373,-2.0428037888,-0.9306762662<br>H,0.3534212589,2.8903756958,-0.7389721194<br>C,0.8850975903,-0.4670295463,-0.9106391918<br>C,-1.2472736586,1.382605536,-0.5267204191<br>C,-0.388266677,-0.8797858106,-0.4496165311<br>C,1.1430377291,0.88373734,-0.2966065637<br>H,2.1767474366,1.2268635308,-0.2574369564<br>H,-1.7738899888,2.1794212886,0.0033538341<br>C,-0.9443120259,0.194279051,0.4541596855<br>H,-2.9615387125,-0.5000819921,0.8028717529<br>C,0.4292362669,0.688365617,1.0997937732<br>C,-2.0860408269,-0.1878425404,1.3792842915<br>H,-0.1829087444,2.7982793906,1.3922410542<br>H,1.2289043978,-1.3277961452,1.5005107225<br>C,0.3094674615,1.9819572243,1.9193717988<br>H,-1.8097147016,-1.0180722445,2.0342835351<br>H,1.3057735746,2.3288119906,2.2108167037	

H,-2.3835937075,0.6584831416,2.0043075578			
C,1.1100368556,-0.3614816364,1.9922060232			
H,2.1037067981,-0.0126140752,2.2902079592			
H,0.5325877928,-0.5192326157,2.9077266811			
H,-0.2506717866,1.7967581382,2.8404710044			
C,0.2578878058,-3.6033488569,-2.5182878157			
H,0.2024918746,-3.412954305,-3.5910481496			
F,-0.7841836161,-4.3765399456,-2.1509293998			
F,1.4131300447,-4.319282829,-2.295678396			
**14** (θ = 179.4 °)			
Electronic Energy= -776.280081248 Hartree			
NIMAG= 0	Atom	Abs.	Rel.
H,-1.2134719292,2.5898893096,-1.1363180992	1H	30.83	1.10
H,1.7158630913,2.0343924919,1.3869886414	2H	24.81	6.93
N,2.0171668363,0.2748517642,0.1861465099	3N	25.38	-176.00
C,1.2480748879,1.2482615291,0.8155289834	4C	56.36	121.42
H,-1.0450507845,0.6129910572,-2.4861423037	5H	30.61	1.31
C,-1.887684404,1.7988442928,-0.8049445549	6C	151.15	30.15
N,1.2554741219,-0.6526628483,-0.4884556002	7N	-50.50	-104.23
H,-2.8999918538,2.2076617564,-0.8138366947	8H	29.76	2.14
C,-0.0466055473,0.9318936998,0.5313397058	9C	48.25	129.24
C,-1.7815094819,0.514376986,-1.6865464408	10C	145.65	35.44
C,0.0276335597,-0.237725944,-0.2700443811	11C	3.70	172.13
C,-1.5061056884,1.2917666378,0.6225942091	12C	129.88	50.63
H,-1.8087871837,1.9481466276,1.4382129457	13H	29.21	2.67
H,-2.7370925688,0.2692378585,-2.1553897345	14H	29.97	1.93
C,-1.3705732476,-0.6237575503,-0.6855475059	15C	125.65	54.70
H,-1.000827936,-2.2227112586,-2.0925992274	16H	30.26	1.65
C,-2.1432662062,-0.1536953065,0.6291562771	17C	115.00	64.95
C,-1.5879296388,-2.0396398231,-1.1882521472	18C	171.54	10.50
H,-4.0420460663,0.3785263261,-0.3825008575	19H	30.78	1.14
H,-0.717785912,-1.0426761454,2.0596473944	20H	31.49	0.46
C,-3.6733547959,-0.1555222798,0.4923395116	21C	163.09	18.64
H,-1.2851854214,-2.7795358337,-0.4428492718	22H	30.71	1.21
H,-4.1245076324,0.3056200253,1.376457348	23H	31.04	0.89
H,-2.6395116621,-2.2102478738,-1.4338321717	24H	30.93	1.00
C,-1.7916722571,-0.9724145575,1.8815632711	25C	161.86	19.83
H,-2.2491586681,-0.5193478822,2.7665124013	26H	31.17	0.77
H,-2.1845014868,-1.9896691192,1.7963080492	27H	31.13	0.80
H,-4.0475740503,-1.1817037912,0.4362819369	28H	30.86	1.07
C,3.418214687,0.1400223542,0.266705122	29C	65.81	112.32
H,3.8470388728,0.9692248048,0.8265061269	30H	25.06	6.69
F,3.9858443345,0.0982552074,-0.9794119756	31F	267.26	-94.21
F,3.7791070311,-1.0381735151,0.8723575325	32F	263.58	-90.68
**14** (θ = 247.1 °)			
Electronic Energy= -776.274682675 Hartree			
NIMAG= 1			
H,0.3495323237,1.8089741177,-2.0867270607			
H,2.1896896935,-1.6227185402,-2.337684058			
N,0.242090844,-2.3612226801,-1.8081778793			
C,1.2942920046,-1.458701689,-1.7593323709			

H,-1.8385395425,1.1169524097,-1.3847613853<br>C,0.2199846505,1.8661181,-1.0050756152<br>N,-0.8120114183,-1.9958788345,-1.0017921884<br>H,0.4333500611,2.8937260969,-0.7042915589<br>C,0.8931655697,-0.4724604329,-0.9042801848<br>C,-1.2050962307,1.4201534408,-0.5491753002<br>C,-0.4007495203,-0.8603498944,-0.4787911382<br>C,1.166691819,0.8650986936,-0.268741719<br>H,2.2066213506,1.1842476621,-0.200850914<br>H,-1.7266558012,2.2221076425,-0.0220486871<br>C,-0.9526306892,0.2149923806,0.4256024918<br>H,-2.9890930853,-0.4481249826,0.7143774975<br>C,0.4154134453,0.6708181558,1.1080941142<br>C,-2.1262115105,-0.1507082697,1.3170389972<br>H,-0.1637724202,2.7892718358,1.4115381132<br>H,1.1628844179,-1.3651120788,1.50652313<br>C,0.3064413881,1.9593122334,1.9376039624<br>H,-1.8828695848,-0.9864592335,1.9782181468<br>H,1.3046739038,2.2857207059,2.245790004<br>H,-2.4297306042,0.6988735103,1.9348667515<br>C,1.0504943156,-0.4022425215,2.0063421585<br>H,2.0423038685,-0.0771427402,2.3357643607<br>H,0.4446161794,-0.5583787973,2.9037167653<br>H,-0.2692369694,1.7759006879,2.8493325699<br>C,0.2813993287,-3.6291236306,-2.4714569973<br>H,1.0661330033,-4.2915046501,-2.1034619692<br>F,0.5236799533,-3.4247994923,-3.8126894143<br>F,-0.9123504039,-4.2458150853,-2.3604233421	
